# Longitudinal analysis of the developing rhesus monkey brain using magnetic resonance imaging: birth to adulthood

**DOI:** 10.1007/s00429-015-1076-x

**Published:** 2015-07-10

**Authors:** Julia A. Scott, David Grayson, Evan Fletcher, Aaron Lee, Melissa D. Bauman, Cynthia Mills Schumann, Michael H. Buonocore, David G. Amaral

**Affiliations:** Department of Neurology, University of California, Davis, Sacramento, USA; Department of Psychiatry and Behavioral Sciences, The MIND Institute, University of California, Davis, 2825 50th Street, Sacramento, CA 95817 USA; Department of Radiology, University of California, Davis, Sacramento, USA

**Keywords:** *Macaca mulatta*, Nonhuman primate, Development, Allometry, Sexual dimorphism

## Abstract

We have longitudinally assessed normative brain growth patterns in naturalistically reared Macaca mulatta monkeys. Postnatal to early adulthood brain development in two cohorts of rhesus monkeys was analyzed using magnetic resonance imaging. Cohort A consisted of 24 rhesus monkeys (12 male, 12 female) and cohort B of 21 monkeys (11 male, 10 female). All subjects were scanned at 1, 4, 8, 13, 26, 39, and 52 weeks; cohort A had additional scans at 156 weeks (3 years) and 260 weeks (5 years). Age-specific segmentation templates were developed for automated volumetric analyses of the T1-weighted magnetic resonance imaging scans. Trajectories of total brain size as well as cerebral and subcortical subdivisions were evaluated over this period. Total brain volume was about 64 % of adult estimates in the 1-week-old monkey. Brain volume of the male subjects was always, on average, larger than the female subjects. While brain volume generally increased between any two imaging time points, there was a transient plateau of brain growth between 26 and 39 weeks in both cohorts of monkeys. The trajectory of enlargement differed across cortical regions with the occipital cortex demonstrating the most idiosyncratic pattern of maturation and the frontal and temporal lobes showing the greatest and most protracted growth. A variety of allometric measurements were also acquired and body weight gain was most closely associated with the rate of brain growth. These findings provide a valuable baseline for the effects of fetal and early postnatal manipulations on the pattern of abnormal brain growth related to neurodevelopmental disorders.

**Electronic supplementary material:**

The online version of this article (doi:10.1007/s00429-015-1076-x) contains supplementary material, which is available to authorized users.

## Introduction

One of the greatest challenges of modern neuroscience is characterizing the dynamic processes of brain maturation and correlating them with the emergence of behavioral and cognitive functions. This is important not only for understanding normal brain development but also for investigating the etiology of neurodevelopmental disorders. Historically, the time course of brain development has been generated through cross-sectional, postmortem studies of animal brains (e.g., Holt et al. 1975; Paxinos et al. 2007) and to a more limited extent of the human brain (Conel 1939; Yakovlev and Lecours 1967). More recently, magnetic resonance imaging (MRI) has allowed in vivo analysis of animal and human brain development (Giedd et al. 1999; Knickmeyer et al. 2010).

Studies of early postnatal brain development in children are still rare but are beginning to appear in the literature (Dean et al. 2014; Fonov et al. 2011; Knickmeyer et al. 2008; Raznahan et al. 2012). A notable contribution is the study by Knickmeyer and colleagues (Knickmeyer et al. 2008), in which children were scanned at 2–4 weeks, 1 year, and 2 years of age. They demonstrated that there was 101 % total brain growth in the first year of life and 15 % growth in the second year. They also demonstrated different trajectories of gray and white matter development. Numerous studies have since been published based on an expansion of this cohort, which have characterized parameters such as local gray matter growth (Gilmore et al. 2012), fiber tract maturation (Geng et al. 2012) and cortical gyrification (Li et al. 2014a, b). As important as these studies are, the intervals between scans were quite large and only 60 % of the children had scans at all three time points.

The non-human primate provides a valuable model for defining the dynamic features of postnatal brain development. Not only can MRI scans be carried out at shorter intervals but the same animals can be scanned throughout development. We have capitalized on the resources of the California National Primate Research Center (CNPRC) which houses approximately 5000 rhesus monkeys and half of these are born and raised in naturalistic outdoor enclosures.

There is a rich history of histological and morphometric studies in non-human primates that have charted brain and body growth from fetal stages to adulthood (Holt et al. 1975; Kerr et al. 1969; Sacher and Staffedlt 1974; Schultz 1933; van Wagenen and Catchpole 1956). These investigations found considerable individual differences in brain size (Holt et al. 1975; Joffe et al. 2005; Kerr et al. 1969) that were associated with differences in body size and sex (Holt et al. 1975; Joffe et al. 2005). Sacher and Staffeldt (1974), for example, carried out a comparative study of the relationship between gestational time, body size and brain weight in different mammals. They found that the rhesus monkey brain is about 68 % of adult size at birth a finding that was confirmed by Holt et al. (1975).

There have been three previous structural MRI studies of the developing rhesus monkey brain (Knickmeyer et al. 2010; Liu et al. 2015; Malkova et al. 2006). These papers have provided important new findings but were limited either in sample size or age range. The Malkova et al.’s study (2006) was based on the observations of seven rhesus monkeys beginning at 1 week of age; four of these animals were imaged longitudinally to 4 years of age. Knickmeyer et al. (2010) studied a substantially larger number of rhesus monkey subjects cross-sectionally from 10 months to 5.3 years of age. A more recent paper by Liu et al. (2015) followed 14 male monkeys longitudinally from 6 to 16 months. Each study added significantly to our understanding of rhesus monkey brain development, but do not characterize the normative regional growth trajectories over the entire developmental period.

For a comprehensive assessment of normal postnatal brain development in rhesus monkeys, one would ideally select naturalistically reared animals of both sexes who would undergo repeated, regular MRI scans starting near birth. To address questions regarding postnatal growth of brain and body in the rhesus monkey, we studied one group of naturalistically reared monkeys (Cohort A) from birth through adulthood and another group of monkeys (Cohort B) from birth through the first year of life. In both groups, magnetic resonance images were collected on each subject at seven time points in the first year. Cohort A subjects received additional scans at 3 and 5 years of age. In the current study, we measured total brain volume (TBV) and adapted a regional parcellation template (Styner et al. 2007) to estimate the maturational trajectories of distinct neuroanatomical regions. This is the largest longitudinal MRI study of rhesus monkey brain development to date. In addition, we constructed average template brains at each study time point using state-of-the-art methodologies (Avants et al. 2011; Klein et al. 2009). To our knowledge, these are the first high-quality monkey MRI reference templates in infant and juvenile development (see Rohlfing et al. 2012). The original MRI scans from these unique cohorts and the template images are available as a resource to the neuroimaging community. They should facilitate future MRI studies of primate neurodevelopment at much earlier ages than was previously possible.

## Materials and methods

All experimental procedures were approved by the University of California, Davis, Institutional Animal Care and Use Committee. The study was carried out in accordance with the National Institute of Health Guide for the Care and Use of Laboratory Animals and developed through consultation with the veterinary staff at the CNPRC.

### Subjects and establishment of cohorts

Two cohorts of *Macaca mulatta* (rhesus) monkeys were developed for this study. In Cohort A, there were originally 28 rhesus monkeys (14 males, 14 females) who were born at the CNPRC in the spring of 2007 (Table 1). Infants were raised by their biological mothers in outdoor, half-acre enclosures that housed 75–120 animals made up of several matrilines. The subjects that made up this cohort were selected from seven different enclosures although as many as six subjects could come from a particular enclosure (see Table 1 for details). Subjects were selected to balance social rank as much as possible and also to have mothers that had a proven history of excellent rearing practices and general health. Subject selection was based on the following characteristics of the mother: (1) rank of matriline (high, *n* = 8; middle, *n* = 9; low, *n* = 10); (2) previous reproductive experience (multiparous, *n* = 25; primiparous, *n* = 3); (3) absence of previous medical problems such as diabetes and arthritis. Social rank of the mother was assessed monthly based on two, 30-min observations by CNPRC behavioral specialists.

**Table 1:** Cohort characteristics

Cohort: birth year	ID and birth order	Sex	Social rank	Enclosure	Matriline
I: 2007	1	Male	Low	5	1
	2	Male	High	3	2
	3	Male	High	1	3
	4	Female	Mid	5	4
	5	Female	Mid	7	5
	6	Male	High	6	6
	7	Female	Mid	3	7
	8	Female	Mid	4	8
	9	Male	Mid	2	9
	10	Female	High	1	3
	11	Male	Mid	2	9
	12	Female	Low	7	10
	13	Female	Low	4	11
	14	Female	Low	5	12
	15	Female	Low	7	10
	16	Female	Mid	5	13
	17	Female	Low	4	14
	18	Female	High	7	15
	19	Female	Mid	2	9
	20	Male	Low	5	16
	21	Male	High	2	17
	22	Male	High	7	18
	23	Male	High	5	19
	24	Female	Low	3	20
	25	Male	High	1	3
	26	Male	Low	2	21
	27	Male	Mid	2	9
	28	Male	Low	4	22
II: 2009	29	Male	Mid	3	23
	30	Male	Mid	4	11
	31	Female	High	3	24
	32	Female	Low	3	25
	33	Male	Mid	2	9
	34	Male	High	7	26
	35	Male	Mid	2	9
	36	Male	Low	2	21
	37	Male	Mid	2	27
	38	Female	Low	3	28
	39	Female	Low	2	29
	40	Female	Low	4	14
	41	Female	Low	5	4
	42	Male	Mid	3	2
	43	Female	High	8	30
	44	Male	Low	8	31
	45	Male	High	4	22
	46	Female	Low	2	32
	47	Male	High	8	33
	48	Male	Mid	8	34
	49	Female	Mid	2	9

Cohort B was established with animals born in 2009. This cohort consisted of 21 rhesus monkeys (10 female, 11 male) that were selected in the same way as cohort A (see Table 1). There were several reasons for carrying out a replication study. First, cohort A animals were imaged on a 3.0T scanner and there was noticeable susceptibility artifact in the temporal lobe and cerebellum. To reduce this problem, we imaged cohort B on a 1.5T scanner which is less prone to susceptibility artifact. Second, we noted a period of no net brain growth between 26 and 39 weeks of age followed by resumed gains from 39 to 52 weeks in the cohort A animals. Since this may signal an important milestone in the development of the primate brain, we thought that it deserved replication. Because mothers from both cohorts were selected from the same field cages, some of the offspring in cohort A were related to participants in cohort B. The closest relations were two sets of siblings (subjects 17 and 40, 19 and 35). Given the small number of siblings and the fact that they were imaged on different scanners, we have not studied familial associations of brain development.

### Assessment of infant health

Infant health was assessed at 1 and 4 weeks of age through a physical examination by an experienced veterinarian. Neurobehavioral tests of reflexes and sensorimotor abilities were also conducted. Subjects were eliminated from the study at 4 weeks of age if there was either: (1) loss in weight, (2) anomalies on MRI, such as edema or enlarged ventricles, or (3) other evidence of compromised health status. Two animals were removed at this time point. Data from these animals were not used and two new animals, selected in the same way, were added to cohort A.

Three subjects (5, 7 and 21) of cohort A were hospitalized for symptoms of dehydration caused by bacterial or parasitic gastrointestinal infection. Treatment included administration of fluids and antibiotics. Subject 21 returned to the study after treatment and remained through the 5-year scan. Subjects 5 and 7 had recurrent bouts of similar illnesses leading to the decision to remove them from the study after the 1 year scan; these animals were not replaced. Subject 8 was removed without replacement from the study at 4 months of age due to non-pathogenic diarrhea that was not responsive to treatment. Subject 22 died from thoracic trauma following a conflict within the troop in 2011. To summarize, 27 subjects of cohort A received scans throughout the first year of life; 25 of these animals received a scan at 3 years and 24 were scanned at 5 years of age. In cohort B, one subject (46) had chronic dehydration in infancy and was removed from the study for treatment. Data for this animal were acquired at 1, 4, and 8 weeks of age. Subject 30 died of causes unrelated to this study after 6 months of age so data are only available through 6 months.

### General imaging procedures

MRI scanning took place at 1, 4, 8, 13, 26, 39, 52 (1 year) weeks of age for both cohorts; cohort A had planned scans at 156 weeks (3 years), and 260 weeks (5 years) of age. Animals were relocated from their home enclosures to temporary indoor housing the day prior to testing. For scans between 1 and 26 weeks of age, infants were removed with their mothers and were housed together in a standard indoor housing cage (61 cm W by 66 cm D by 81 cm H). On the day of MRI scanning, the mothers were sedated using ketamine (7–8 mg/kg, IM) and the infants were transferred to an incubator. At the CNPRC, infants are typically weaned at approximately 26 weeks of age. Therefore, beginning at 39 weeks of age, infants were removed from their home enclosures without their mothers and were temporarily housed indoors as described above.

Infants were fasted a minimum of 2 h prior to sedation. They were transported from the CNPRC to the Imaging Research Center (IRC) in either an incubator (30.5 cm W by 30.5 cm D by 30.5 cm H) (1–13 weeks of age) or transport box (31.0 cm W by 51.0 cm D by 40.0 cm H) (26–52 weeks of age). At the IRC, infants were sedated with ketamine (1 mg/kg IM) and intubated with an endotracheal tube (2.0–2.5 mm uncuffed, 3.0–3.5 mm cuffed). Infants were anesthetized with propofol (~2 ml/kg/h IV). Intravenous saline was administered throughout the scanning session. Heart rate and oxygen saturation were monitored in the control room of the scanner suite on a Nonin 8600 pulse oximeter (Nonin Medical, Inc., Plymouth, MN, USA). Anesthetic levels were increased or decreased remotely using a Harvard Apparatus 4500 infusion pump (Harvard Apparatus, Holliston, MA, USA) to maintain stable anesthesia. The animal was also monitored by video camera for the duration of the scan.

For cohort A, the animal was positioned supine on the scanner bed and the head was centered in the head coil of the scanner. In cohort B, the animal was oriented prone and the head was stabilized in a stereotaxic apparatus (Crist Instruments, Hagerstown, MD, USA). Body temperature was maintained by surrounding the animal with heated saline packs and blankets. Oxygen was delivered around the nose and mouth at a rate of 0.5–1.0 L/h to maintain oxygen saturation.

After completion of the scans, propofol delivery was stopped. The total time of sedation ranged from 60 to 90 min. During recovery from sedation, the infants were given subcutaneous saline with 5 % dextrose to maintain hydration and normal blood sugar levels. Infants were also provided access to glucose-enriched water in their incubators. Infants were transported back to the CNPRC following the scan. The younger animals were first reunited with their mothers in holding cages and then returned together to their home enclosures; the older animals were returned directly to their home enclosures. All mothers immediately accepted their infants on each of the reunions. While the capture and anesthetic procedures used in this study undoubtedly induced some stress in the mothers and infants, these procedures are identical to those that are normally carried out for periodic health assessments of all animals at the CNPRC. Therefore, all of the mothers in this study had been exposed to similar procedures many times during routine medical evaluations.

### Specific neuroimaging procedures

All cohort A subjects were imaged on a Siemen’s 3T Trio MRI system using an 8-channel RF head array coil (Invivo, Inc., Gainesville, FL, USA). A 3D T1-weighted MP-RAGE sequence was collected in the sagittal plane (number of slices = 192; slice thickness = 0.7 mm; number of excitations = 1; repetition time = 2200 ms; echo time = 4.73 ms; inversion time = 1100 ms; flip angle = 7°; field of view = 180 mm × 180 mm; matrix = 256 × 256; bandwidth = 140 hertz/pixel). Additional sequences were acquired but are not reported in this paper. Subject 15 missed the 39-week time point due to scanner down time and data were lost for subject 7 at 52 weeks of age.

In cohort B, images were acquired on a 1.5T GE Genesis Signa MRI system with a three inch surface coil. A 3D T1-weighted SPGR with fat saturation sequence was collected in the transverse plane (number of slices = 192; slice thickness = 0.7 mm; number of excitations = 2; repetition time = 27 ms; echo time = 6 ms; flip angle = 30°; field of view = 160 mm × 160 mm; matrix = 256 × 256; bandwidth = 122 hertz/pixel). DTI and T2-weighted sequences were also collected. Total scan time was approximately 45 min.

### Image analysis

#### Age-specific average brain templates

**Fig. 1:**
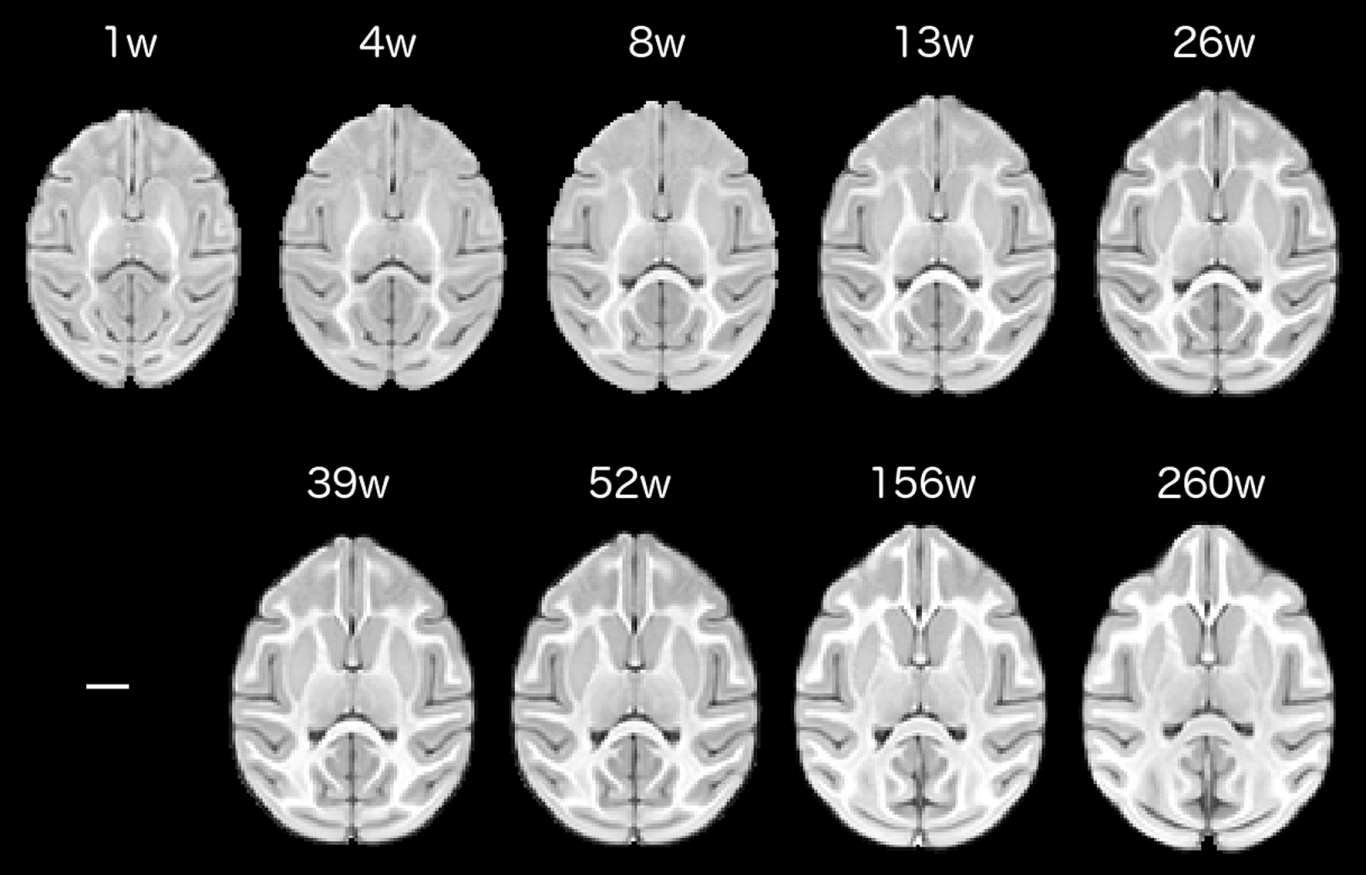
Age-specific, horizontally oriented templates are shown for each study time point. Each template represents an average brain image constructed from the individual subject scans. Images display enhanced signal-to-noise and optimal shape characteristics relative to individual scans. *Scale bar* represents 1 cm

All image processing and preprocessing were carried out using the FSL suite (version 5.0; http://www.fmrib.ox.ac.uk/fsl/) and ANTS suite (version 1.9.x; http://stnava.github.io/ANTs/) of image processing and normalization tools. To facilitate the extraction of reliable morphometric data and to provide a common reference space for group-level analyses, study-specific average T1w template images were constructed for each age from the available individual subject scans. Template construction occurred successively, beginning with the oldest available age (260 weeks), with each template serving as the reference for the construction of the preceding earlier age, until finally the earliest template (1 week) was produced. The overall process of template construction was identical for all ages, so we will describe this process in detail just for the oldest time point. Horizontal template images at each age are shown in Fig. 1.

First, all subject images were rigid-body aligned to the publicly available inia19 brain template (Rohlfing et al. 2012) using FSL’s FLIRT so that all image processing began within a standardized coordinate space. The registration parameters for this step were obtained using the skull-stripped subject images and template, and applied to both the skull-stripped images and whole-head (unstripped) images. The aligned skull-stripped images were then averaged, producing a rigid-body average brain template. The aligned, skull-stripped images were then transformed onto the rigid-body template using 12-parameter affine registration (FLIRT). These parameters were applied to the whole-head images, which were then averaged, producing an affine average whole-head template. This affine average image was used as the reference image for producing a fully deformable template. Though the use of skull-stripped images in linear registration procedures (i.e., rigid-body and 12-parameter) substantially improves the stability of these operations, the use of the whole-head images for the fully deformable templates is more appropriate. Whole-head templates prevent the introduction of any artificial boundaries between brain and skull, and ultimately provide a more useful analytic resource.

The fully deformable template was built from the whole-head images using the default settings in the ‘buildtemplateparallel.sh’ script provided in the ANTS package, which proceeds according to the well-validated “optimal shape” methodology laid out in detail in Avants et al. (2011). Briefly, a template is constructed by iteratively averaging subject images together, then computing the linear and nonlinear transformations to map subjects onto the average, obtaining a new template by averaging the transformed images, re-computing the transformations to the new template, and so forth. The linear affine matrices and nonlinear deformations were obtained using the ANTS-SyN symmetric normalization algorithm with correlation coefficient cost-function (Avants et al. 2011). After each iteration of averaging, the template was also updated to ensure average shape characteristics by applying the inverse of the average of the subject-to-template warps. A final template image was produced after four iterations of deformable registrations and averaging.

For all ages other than 260 weeks, the entire process was identical except that subjects were initially rigid-body aligned to the template that was older by one step rather than the inia19 image itself. This allowed all age-specific templates to be maintained in the inia19 coordinate space (since all templates are aligned successively), while minimizing the problem of aligning images with vastly different contrast features. Likewise, construction of templates for each time point of the cohort B study used the corresponding age-specific template from the cohort A study as a starting reference. We note, however, that the starting reference image only determines the coordinate space in which the study-specific templates are built (inia19 space), and does not impact the construction or the features of the template image itself.

#### Mapping the anatomical parcellation across ages

We obtained the fully deformable parameters for spatially transforming age-specific templates to each other using the ANTS-SyN symmetric normalization algorithm provided in the ANTS software package. Specifically, each template was registered onto the older age template using the ‘ANTS’ command with an iterative scheme of 50 × 90 × 30 (50 iterations at the coarsest resolution, 90 at the next highest resolution, 30 at the finest resolution). This scheme ensured that transforming images with stable contrast (all time points at 26 weeks and later) reached convergence reliably and quickly. Furthermore, we confirmed visually that for this subset of registrations, each transformed image was indistinguishable from its reference image.

A whole-brain anatomical parcellation was defined on the 260-week template image according to the guidelines listed below in “Definition of anatomical regions”. This parcellation was then mapped to each age-specific template by concatenating all the necessary stepwise transformations. For instance, to map the parcellation down to the 52-week template, we concatenated the 260–156 and 156–52 weeks transformations and applied the combined transformation to the 260-week parcellation. The parcellation was interpolated with a “multilabel” solution, which consisted first of nearest-neighbor interpolation followed by Gaussian smoothing of each label with sigma equal to the image voxel size, followed by a winner-take-all voting scheme to assign each voxel its final label. To map the parcellation onto the 1 week template, we concatenated all the stepwise transformations and repeated the same procedure. For ages 13 weeks and earlier, the parcellations were manually inspected and edited for accuracy.

Parcellations for the cohort B study were defined similarly. Each age-specific template from the cohort A study was transformed to the corresponding template in the cohort B study using the ANTS command with the 50 × 90 × 30 iterative scheme. The deformable parameters obtained from this process were then applied to the edited age-specific parcellation from the cohort A study and interpolated on the cohort B template images using the multilabel solution as just described. No further editing was required on the cohort B templates. Individual subject parcellations were defined at each age by simply applying the inverse of subject-to-template transformations (as obtained during template construction) to each age-specific parcellation.

#### Definition of anatomical regions

**Fig. 2:**
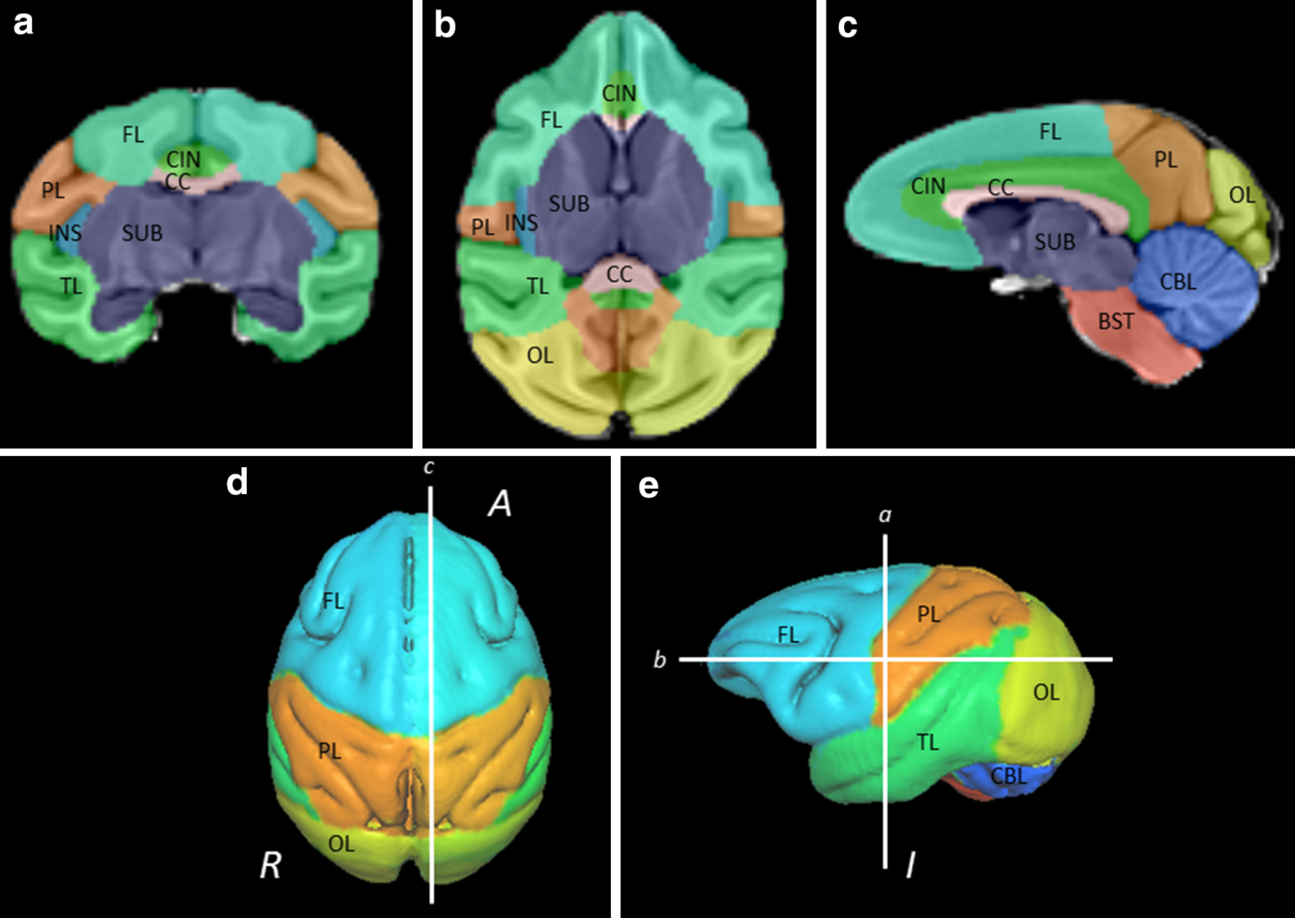
The 5 year-old template image with overlaid parcellations shown in (**a**) coronal, (**b**) axial, and (**c**) sagittal cross sections and surface renderings with (**d**) superior and (**e**) left lateral views. ROIs shown are frontal lobe (*FL*), parietal lobe (*PL*), temporal lobe (*TL*), occipital lobe (*OL*), insula (*INS*), cingulate (*CIN*), corpus callosum (*CC*), subcortical structures (*SUB*), brain stem (*BST*), and cerebellum (*CBL*)

The current definition of anatomical regions was based on the validated parcellation atlas developed by Styner and colleagues (Knickmeyer et al. 2010; Styner et al. 2007) with modifications (Fig. 2). The original atlas was registered to our 260-week template image prior to making any modifications. Then, the regional boundaries were reviewed and edited. For our analysis, we consolidated subregions of the temporal and frontal lobes. We also removed the hippocampal region and amygdala from the definition of the temporal lobe and included these regions in the subcortical ROI. Lobar boundaries were guided by rhesus monkey histological atlases (scalablebrainatlas.incf.org/cocomac/) (Felleman and Van Essen 1991; Paxinos et al. 1999). For our parcellation, the following regions were defined: right and left occipital (OL), parietal (PL), temporal (TL), frontal (FL), cingulate (CIN), and insular (INS) regions; right and left cerebellum (CB); corpus callosum (CC), subcortical structures (SUB), and brain stem (BST). The TBV that we report is the sum of these regions of interest. Details concerning modifications from the Styner et al. (2007) parcellation are available from the authors.

### Physical growth measurements

On the same day as the MRI sessions, several measures of body growth were collected. Body measurements included weight, crown-rump length, circumferences of the head, chest, and right arm, occipito-frontal and bi-parietal diameters of the head, and lengths of the right hand, foot, and femur. Crown-rump length was measured as the distance from the top of the head to the base of the tail. Head circumference was measured around the widest portion of the skull. Chest circumference was measured at the level of the nipples. Arm circumference was measured around the largest part of the bicep. Occipito-frontal diameter was the maximum distance from the frontal bone to the occipital bone and the bi-parietal diameter was the maximum distance from one parietal eminence to the other. Hand length was measured from the junction of the hand and wrist to the length of the third digit. Similarly, foot length was the distance from the heel to the longest toe. Femur length was the distance from the hip joint to the distal end at the knee. All measurements were taken twice and the average was used for analysis. The repeated measurements differed by less than 5 %. If, upon analysis, a data point was found to be in error (e.g., a length measure being greater at an earlier time point), it was excluded from the dataset. This occurred no more than once for a particular measurement at any particular time point. For cohort A, all measurements were collected from 1 to 156 weeks. For cohort B, only crown-rump length, head circumference, bi-parietal diameter, occipito-frontal diameter, and weight were collected at each time point (1 through 52 weeks).

### Statistical analysis

All statistical analyses were carried out using SPSS 21.0 software (SPSS Inc., Chicago, IL, USA). Linear mixed models were used to estimate growth trajectories for body growth parameters over the 1–156 weeks age range. Separate analyses of the same type were carried out for each cohort. For TBV and body weight, the age range extended to 260 weeks of age. ROI volumes were calculated only for the 24 subjects with a 260-week scan and, therefore, these trajectories are based on a subset of data points. Linear mixed models with natural log transformed age were used in these models since the age-related changes were not linear. First, second, and third order terms were tested to model the growth trajectories of brain regions. Parameters such as sex and hemisphere were added to the model where appropriate. Growth curves from 1 to 52 weeks of age were independently tested for the 21 subjects of the second cohort. We used a linear mixed model to test for potential differences in cohort characteristics in TBV. To compare relative growth rates of the lobar regions, relative growth parameters were calculated by dividing the volume at each time point by the volume at 1 week of age.

Observation of the raw data suggested that there was little to no change in brain volume between 26 and 39 weeks of age. To specifically test the change around 39 weeks, repeated measures ANOVA tests including 26, 39, and 52 weeks time points were conducted for TBV, cerebral volume, and cerebellar volume.

## Results

We begin our description by presenting the MRI data from cohort A that has the most comprehensive dataset on brain development. Because we found reliable sex differences in the volume of the brain, we will report data for males and females independently. We then present the allometric results and the relationships between body and brain growth. Finally, we compare the findings in cohorts A and B.

### Total brain volume (Table 2)

The mean TBV for the male subjects of cohort A at 5 years of age was 98,653 mm^3^ (±5258 mm^3^). When these animals were 1 week of age, their mean TBV was 62,379 mm^3^ (±4398 mm^3^) or 63 % of the adult size. For the females, the TBV at 5 years was 91,283 mm^3^ (±4314 mm^3^) and at 1 week was 59,929 mm^3^ (±2408 mm^3^) or 65 % of the adult size. Thus, there was substantial postnatal growth of the brain. On average, male brains were larger than female brains at every time point (*p* < 0.05). The sex difference in TBV was on the order of 5 % up to 26 weeks and increased to 8 % for subsequent ages.

**Table 2:** Bilateral brain volumes

	Age (weeks)								
	1	4	8	13	26	39	52	156	260
Female									
Total brain	59,929 (2408)	68,932 (2916)	76,313 (3282)	80,876 (3687)	85,224 (4184)	83,298 (3784)	86,564 (4500)	91,625 (3835)	91,283 (4314)
Cerebrum	54,856 (2328)	62,948 (2810)	69,367 (3090)	73,154 (3390)	76,665 (3813)	74,525 (3518)	77,244 (4095)	80,701 (3580)	79,966 (3944)
Cerebellum	4093 (292)	4884 (339)	5706 (433)	6332 (501)	6929 (563)	7012 (525)	7404 (585)	8452 (486)	8540 (569)
Brain stem	980 (36)	1100 (43)	1241 (57)	1390 (68)	1630 (83)	1761 (84)	1915 (96)	2472 (109)	2777 (139)
Corpus callosum	434 (22)	529 (40)	637 (41)	735 (54)	901 (63)	930 (72)	940 (69)	1151 (83)	1255 (89)
Subcortical structures	7408 (181)	8109 (223)	8738 (294)	9355 (337)	10,319 (387)	10,554 (340)	11,188 (491)	12,481 (406)	12,580 (490)
Male									
Total brain	62,379 (4398)	72,212 (4839)	78,989 (5253)	84,045 (5070)	88,649 (5102)	90,065 (5258)	91,737 (6114)	98,027 (5563)	98,653 (5258)
Cerebrum	56,758 (4023)	65,511 (4409)	71,355 (4828)	75,565 (4705)	79,205 (4786)	80,105 (4913)	81,286 (5761)	85,649 (5154)	85,568 (4640)
Cerebellum	4590 (490)	5533 (633)	6333 (718)	7013 (740)	7728 (772)	8051 (776)	8377 (796)	9631 (883)	9908 (925)
Brain stem	1031 (70)	1168 (84)	1300 (93)	1467 (103)	1716 (122)	1910 (139)	2074 (161)	2747 (226)	3178 (282)
Corpus callosum	442 (36)	544 (43)	643 (51)	748 (68)	915 (77)	974 (74)	983 (84)	1214 (108)	1361 (134)
Subcortical structures	7559 (460)	8322 (522)	8905 (567)	9557 (616)	10,556 (638	11,136 (665)	11,616 (735)	13,201 (786)	13,499 (832)

Mean (standard deviation) in mm^3^

**Fig. 3:**
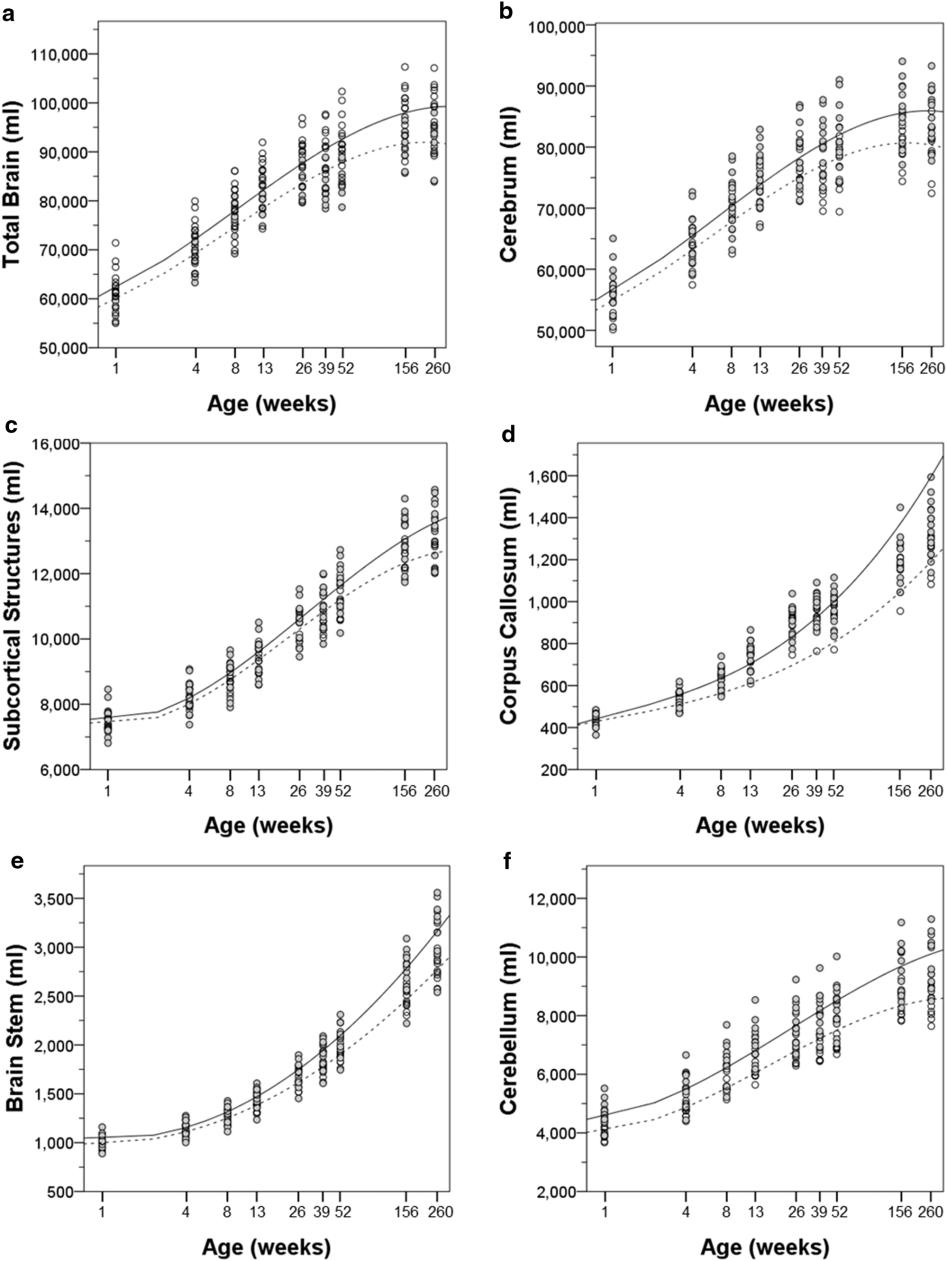
Growth trajectories of bilateral brain structures for male (*solid*) and female (*dashed*) subjects from 1 to 260 weeks of age. **a** Total brain, **b** cerebrum, **c** subcortical structures, **d** corpus callosum, **e** brain stem, **f** cerebellum

As described in “Materials and methods”, MRIs were acquired at 1, 4, 8, 13, 26, 39, 52, 156 and 260 weeks of age. We found that brain volume at 1 week of age predicted the TBV growth rate (*F* = 11.317, *p* = 0.001). TBV increased over each measurement interval with the exception of the periods between 26 and 39 weeks of age (*t* = −0.449, *p* = 0.658) and 156 to 260 weeks (*t* = −0.631, *p* = 0.534) (Table 2; Fig. 3). The most rapid rate of growth occurred between 1 and 4 weeks and decreased at each of the measured intervals thereafter (Table 3).

**Table 3:** Total brain growth rates calculated between time points

	Weekly rate of TBV change (mm^3^/week)^a^							
	1–4	4–8	8–13	13–26	26–39	39–52	52–156	156–260
Female	3001 (484)	1845 (203)	913 (228)	334 (94)	−101 (181)	200 (161)	49 (16)	−3 (9)
Male	3278 (515)	1694 (354)	1011 (177)	354 (109)	109 (137)	129 (134)	60 (25)	6 (15)

^a^Mean (standard deviation) in mm^3^

Interestingly, the TBV growth rate plateaued between 26 and 39 weeks (*t* = −0.449, *p* = 0.658) (Table 3; Fig. 3a). For females, there was actually a negative growth rate during this period and the average TBV decreased from 85,224 mm^3^ (±4184 mm^3^) to 83,298 mm^3^ (±3784 mm^3^). The growth rate for the males during this period was positive but lower than the rate for the period between 13 and 26 weeks and for the period from 39 to 52. In fact, positive gains in volume resumed in all subjects from 39 to 52 weeks of age (*t* = −5.193, *p* < 0.001) (Table 3).

The change from 3 to 5 years revealed a sex difference in growth rates. Females had achieved a maximal TBV by 3 years and had a modest decline (−3.28 mm^3^/week) to the 5-year time point, while males showed a TBV increase of 6.02 mm^3^/week. These differences suggest a longer maturational period in males.

A growth trajectory model was developed based on the data that have been acquired at the fixed time points in this study. The best fit for TBV required a third-order polynomial with log transformed age (cubic term, *F* = 35.696, *p* < 0.001) (Table 4). The model estimated a 47 % increase in female and 53 % increase in male brain volume from 1 to 52 weeks of age, which slightly overestimates the actual mean increases of 44 and 47 %, respectively. Over the full 5 years, the estimated gain in TBV was 49 % in females and 60 % in males, while the actual gains were 52 and 58 %, respectively. The correspondence between the real data and the model supports the fit of the cubic natural log transformed trajectory (Table 4). The growth curve for TBV in males was determined to be:

$${\text{TBV}} = 5900 \times \ln ({\text{age}}) + 1242 \times \left( {\ln ({\text{age}})} \right)^{2} - 199 \times \left( {\ln ({\text{age}})} \right)^{3} +\,62{,}114$$

and for females:

$${\text{TBV}} = 5406 \times \ln ({\text{age}}) + 1300 \times \left( {\ln ({\text{age}})} \right)^{2} - 222 \times \left( {\ln ({\text{age}})} \right)^{3} + \, 59{,}807.$$

The intercept predicts the brain volume at birth and the parameter estimates predict the age-related changes in volume.

**Table 4:** Growth trajectory estimates, brain bilateral regions

	Linear		Quadratic		Cubic		Intercept	
Female								
Total brain	5406 (897)***		1301 (458)**		−223 (56)***		59,807 (1158)***	
Cerebrum	5161 (818)***		939 (418)*		−187 (51)***		54,746 (1069)***	
Subcortical structures	−80 (88)		467 (45)***		−51 (6)***		7358 (114)***	
Corpus callosum	13 (16)		40 (8)***		3 (1)***		429 (20)***	
Brain stem	13 (8)		55 (1)***				987 (29)***	
Male								
Total brain	5900 (695)***		1243 (360)**		−199 (44)***		62,113 (1386)***	
Cerebrum	5505 (627)***		923 (325)**		−173 (40)***		56,515 (1258)***	
Subcortical structures	−22 (83)		443 (43)***		−44 (5)***		7490 (184)***	
Corpus callosum	27 (16)		49 (8)***		4 (1)***		441 (25)***	
Brain stem	−13 (12)		71 (2)***				1036 (53)***	

Parameter estimates (SE) in mm^3^ for linear, quadratic and cubic term in LMM with ln(age)

* *p* < 0.05, ** *p* < 0.01, *** *p* < 0.001 for LMM with ln(age) by sex

### Regional volumes: cortical

We now turn to a description of the parcellated regions of the brain with a focus on the cerebral cortex (Tables 2, 5, 6). As expected, the growth trajectory of the cerebrum (all neocortical regions bilaterally) closely matched that of the total brain (Table 4; Fig. 3b). As with TBV, the cerebrum showed no significant change in volume between 26 and 39 weeks (*t* = −0.386, *p* = 0.703) and between 3 and 5 years of age (*t* = −1.514, *p* = 0.144). For most regions within the cerebrum, male volumes were larger than corresponding female volumes (*p* < 0.001). Interestingly, the only exception was the frontal lobe, which did not differ by sex (*F* = 2.67, *p* = 0.115).

**Table 5:** Regional brain volumes, right hemisphere

	Age (weeks)								
	1	4	8	13	26	39	52	156	260
Female									
Frontal lobe	6315 (371)	7128 (421)	79,000 (465)	8505 (482)	9179 (530)	8973 (372)	9519 (564)	10,091 (580)	9894 (664)
Parietal lobe	4460 (281)	5160 (332)	5683 (340)	5958 (354)	6160 (394)	5836 (353)	6030 (396)	6047 (394)	6009 (439)
Temporal lobe	5847 (284)	6681 (336)	7585 (405)	8025 (465)	8504 (522)	8342 (510)	8684 (520)	8935 (537)	8757 (513)
Occipital lobe	5665 (355)	6650 (473)	7249 (518)	7336 (568)	7063 (633)	6571 (663)	6526 (663)	6482 (587)	6392 (585)
Cingulate cortex	887 (46)	975 (59)	1124 (67)	1177 (74)	1221 (74)	1190 (63)	1252 (64)	1301 (70)	1257 (57)
Insular cortex	380 (27)	424 (28)	442 (26)	448 (33)	445 (35)	440 (38)	453 (30)	477 (34)	482 (45)
Cerebellum	1974 (143)	2358 (168)	2725 (236)	3061 (232)	3350 (269)	3389 (249)	3585 (281)	4097 (225)	4136 (257)
Male									
Frontal lobe	6471 (567)	7364 (664)	8031 (680)	8682 (710)	9370 (786)	9641 (763)	9899 (755)	10,552 (838)	10,405 (748)
Parietal lobe	4660 (422)	5421 (479)	5895 (555)	6231 (561)	6432 (582)	6406 (575)	6389 (697)	6497 (576)	6486 (482)
Temporal lobe	6113 (443)	7060 (457)	7803 (505)	8327 (475)	8886 (463)	8981 (461)	9141 (638)	9595 (471)	9529 (407)
Occipital lobe	5916 (435)	6955 (534)	7475 (596)	7602 (553)	7310 (577)	7083 (643)	6957 (706)	6833 (604)	6734 (572)
Cingulate cortex	918 (63)	1038 (61)	1186 (82)	1246 (76)	1283 (76)	1302 (72)	1331 (113)	1374 (72)	1366 (88)
Insular cortex	394 (31)	446 (38)	457 (40)	467 (39)	463 (38)	472 (31)	481 (40)	502 (39)	523 (46)
Cerebellum	2210 (240)	2666 (306)	3060 (355)	3392 (355)	3730 (378)	3889 (368)	4061 (395)	4663 (418)	4793 (445)

Mean (standard deviation) in mm^3^

**Table 6:** Regional brain volumes, left hemisphere

	Age (weeks)								
	1	4	8	13	26	39	52	156	260
Female									
Frontal lobe	6414 (365)	7404 (409)	8246 (428)	8847 (476)	9531 (506)	9299 (371)	9788 (585)	10,387 (518)	10,211 (615)
Parietal lobe	4574 (309)	5299 (339)	5807 (369)	6090 (373)	6308 (387)	6026 (412)	6164 (417)	6270 (381)	6189 (429)
Temporal lobe	5730 (330)	6641 (357)	7401 (393)	7897 (439)	8514 (526)	8315 (530)	8549 (536)	8965 (528)	8859 (505)
Occipital lobe	5574 (360)	6604 (491)	7174 (526)	7323 (575)	7033 (607)	6580 (631)	6630 (659)	6561 (569)	6509 (566)
Cingulate cortex	799 (41)	936 (55)	956 (58)	1017 (58)	1046 (67)	1029 (60)	1052 (54)	1074 (52)	1079 (61)
Insular cortex	369 (22)	408 (21)	426 (26)	440 (26)	443 (31)	441 (32)	471 (40)	480 (32)	492 (48)
Cerebellum	2119 (152)	2526 (175)	2981 (231)	3271 (273)	3578 (296)	3624 (279)	3820 (308)	4355 (263)	4405 (316)
Male									
Frontal lobe	6545 (534)	7580 (639)	8423 (667)	9055 (696)	9744 (754)	9973 (734)	10,226 (840)	10,908 (812)	10,797 (748)
Parietal lobe	4711 (485)	5500 (514)	5990 (583)	6312 (602)	6536 (625)	6547 (644)	6605 (690)	6694 (609)	6618 (530)
Temporal lobe	5969 (495)	6928 (477)	7661 (563)	8213 (535)	8804 (492)	8853 (518)	9036 (508)	9565 (539)	9578 (477)
Occipital lobe	5852 (453)	6936 (499)	7439 (591)	7612 (564)	7350 (571)	7152 (625)	7002 (771)	7050 (582)	6958 (535)
Cingulate cortex	822 (69)	992 (72)	1002 (80)	1055 (78)	1096 (87)	1110 (85)	1125 (72)	1153 (97)	1191 (78)
Insular cortex	385 (33)	427 (36)	446 (43)	456 (37)	461 (42)	476 (36)	495 (36)	512 (38)	524 (49)
Cerebellum	2381 (251)	2867 (328)	3273 (367)	3622 (388)	3997 (397)	4162 (410)	4316 (403)	4969 (467)	5115 (482)

Mean (standard deviation) in mm^3^

**Fig. 4:**
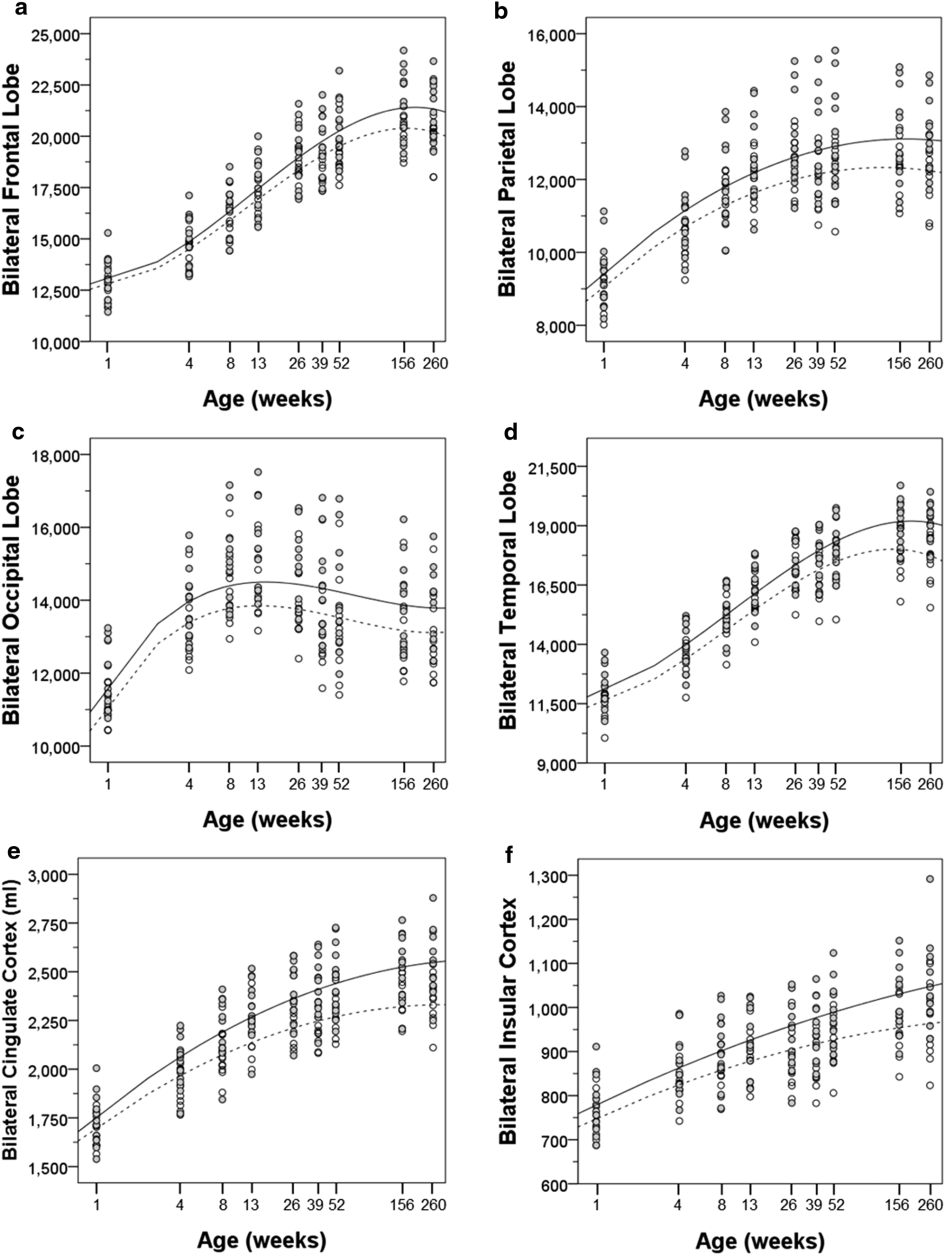
Bilateral regional cortical volumes and growth trajectories are plotted for male (*solid*) and female (*dashed*) subjects. **a** Frontal lobe, **b** parietal lobe, **c** temporal lobe, **d** occipital lobe, **e** cingulate cortex, and **f** insular cortex

**Fig. 5:**
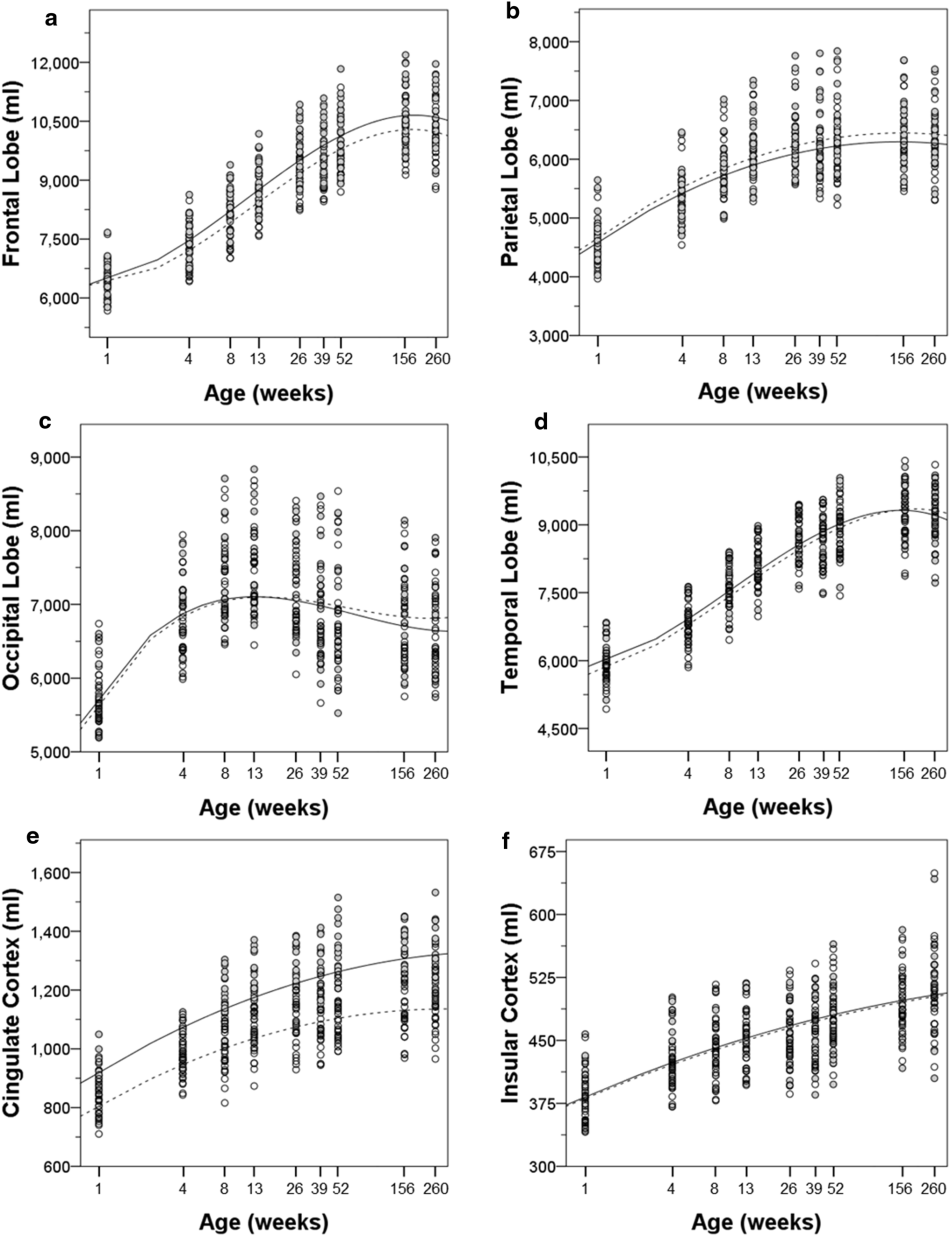
Regional cortical volumes and growth trajectories are plotted for left (*solid*) and right (*dashed*) hemispheres. **a** Frontal lobe, **b** parietal lobe, **c** temporal lobe, **d** occipital lobe, **e** cingulate cortex, and **f** insular cortex

Cortical regions differed in the time course of volume changes over the lifespan. The growth curves of each bilateral cortical region are illustrated in Fig. 4 and the hemispheric cortical regions are illustrated in Fig. 5. The parameter estimates for the log transformed polynomial curves are provided in Tables 4, 7 and 8.

**Table 7:** Growth trajectory estimates, right hemisphere regions

	Linear	Quadratic	Cubic	Intercept
Female				
Frontal lobe	397 (109)***	300 (56)***	−44 (7)***	6378 (155)***
Parietal lobe	682 (31)***	−70 (8)***		4589 (116)***
Temporal lobe	454 (102)***	211 (52)***	−34 (6)***	5699 (140)***
Occipital lobe	1225 (105)***	−344 (54)***	28 (54)***	5577 (163)***
Cingulate cortex	107 (8)***	−10 (1)***		796 (16)***
Insular cortex	31 (4)***	−2 (1)**		373 (10)***
Cerebellum	127 (48)*	155 (25)***	−18 (3)***	2097 (78)***
Male				
Frontal lobe	388 (89)***	329 (46)***	−46 (6)***	6503 (187)***
Parietal lobe	731 (36)***	−72 (6)***		4755 (148)***
Temporal lobe	492 (69)***	202 (36)***	−31 (4)***	5942 (138)***
Occipital lobe	1230 (95)***	−333 (49)***	27 (6)***	5825 (153)***
Cingulate cortex	121 (10)***	−10 (2)***		816 (22)***
Insular cortex	32 (4)***	−2 (1)*		388 (11)***
Cerebellum	185 (40)***	144 (21)***	−16 (3)***	2349 (107)***

Parameter estimates (SE) in mm^3^ for linear, quadratic and cubic term in LMM with ln(age)

* *p* < 0.05, ** *p* < 0.01, *** *p* < 0.001 for LMM with ln(age) by sex and hemisphere

**Table 8:** Growth trajectory estimates, left hemisphere regions

	Linear	Quadratic	Cubic	Intercept
Female				
Frontal lobe	192.5 (106.0)***	360.200 (54.5)***	−49.7 (6.7)***	6284.2 (161.2)***
Parietal lobe	677.4 (50.5)***	−72.700 (50.5)***		4496.7 (113.5)***
Temporal lobe	379.9 (102.5)***	249.400 (52.4)***	−40.1 (6.41)**	5853.0 (137.5)***
Occipital lobe	1189.7 (114.2)***	−340.600 (58.7)***	27.6 (7.2)***	5643.6 (168.4)***
Cingulate cortex	119.9 (13.3)***	−9.400 (2.3)***		896.8 (21.1)***
Insular cortex	30.3 (4.0)***	−1.9000 (0.7)**		375.0 (10.5)***
Cerebellum	110.6 (54.9)*	147.800 (28.0)***	−17.3 (3.4)***	1965.2 (69.1)***
Male				
Frontal lobe	229.2 (76.7)***	360.100 (39.9)***	−48.9 (4.9)***	6427.4 (188.8)***
Parietal lobe	693.9 (42.7)***	−66.300 (7.3)***		4684.3 (137.1)***
Temporal lobe	466.3 (82.5)***	217.000 (42.4)***	−33.9 (5.2)**	6095.5 (127.3)***
Occipital lobe	1211.5 (94.6)***	−334.400 (48.8)***	26.0 (6.0)***	5912.2 (154.3)***
Cingulate cortex	129.7 (11.3)***	−9.400 (1.9)***		936.5 (22.5)***
Insular cortex	32.8 (4.6)***	−1.8000 (0.8)*		390.7 (10.7)***
Cerebellum	169.7 (40.6)***	139.500 (21.1)***	−15.3 (2.6)***	2190.9 (99.6)***

Parameter estimates (SE) in mm^3^ for linear, quadratic and cubic term in LMM with ln(age)

* *p* < 0.05, ** *p* < 0.01, *** *p* < 0.001 for LMM with ln(age) by sex and hemisphere

There was rapid growth of the frontal lobe in both males and females in the first 3 months of life. Between 26 and 39 weeks, there was a slight decrease in the volume of the frontal lobe in the females (right −8.00 ± 20.86 mm^3^/week, left −9.69 ± 21.58 mm^3^/week), whereas it continued to grow at a slower pace in the males. In both males and females, the frontal lobe reached its largest extent by 3 years and slightly decreased in size by 5 years. The trajectory of temporal lobe growth was very similar to that of the frontal lobe (see Fig. 5).

The parietal lobe growth trajectory was somewhat different from the frontal and temporal lobes. The pattern was similar up to 9 months of age including the decrease in volume in the females between 26 and 39 weeks. However, a near maximal volume was achieved by 39 weeks in the males and 52 weeks in the females with little or no gain thereafter. The pattern of cingulate growth was very similar to that of the parietal lobe.

The insular cortex had a flatter growth trajectory that nonetheless continued throughout the 5-year period.

The trajectory of occipital lobe growth was the most different from the other cortical regions. The occipital lobe underwent the most rapid growth of any cortical region between 1 and 4 weeks of age (right 338.07 ± 66.13 mm^3^/week, left 353.01 ± 60.54 mm^3^/week). The volume of the occipital lobe peaked at 13 weeks of age, followed by a continuous decline through 5 years of age. Thus, the volume of the occipital lobe of the 5-year-old animals was similar to that at 4 weeks of age.

The growth trajectory of the corpus callosum (Fig. 3d) is partially reminiscent of that of the cerebrum. There was rapid growth between birth and 39 weeks in both males and females. There was then a plateau in growth rate between 39 and 52 weeks followed by continued growth. The plateau occurred later than in cortical regions as described above. One other distinction from cortical growth was that the callosum volume continued to increase throughout the 5 years in both males and females. There was no significant effect of sex for the corpus callosum.

### Regional volumes: subcortical

Due to the difficulty in reliably parcellating small subcortical regions during development based on MRI scans, we have combined a number of brains regions into either “subcortical structures” or brainstem. Both these regions increased in volume throughout the analysis period (Fig. 3c, e). Unlike the cortical regions, there was no plateau in growth between 26 and 39 weeks of age.

The cerebellum was larger in the males than in the females at all time points (Fig. 3f). The cerebellum’s growth trajectory was different from that of the cerebrum in two ways. First, there was no plateau in growth during the 26–39 weeks period. Secondly, there was continued, albeit modest, increase in cerebellar volume between 3 and 5 years. The growth curves suggested that male volume may be continuing beyond this time point whereas volume increases in the females had certainly tapered off between 3 and 5 years.

### Percentage of postnatal growth

In addition to describing the growth trajectories of brain regions, it is also informative to summarize the percentage change in volume from 1 to 260 weeks for different cortical and subcortical brain regions. Within the cerebral cortex, the frontal and temporal lobes showed the greatest degree of growth, exceeding 50 % gain over this age range. On average, the parietal lobe (37 %), cingulate cortex (46 %), and insular cortex (30 %) showed less volume increases. Since the trajectory of the occipital lobe demonstrated a much earlier peak than other regions followed by a sharp decline, it is important to highlight the relative change through each of the phases. The occipital lobe increased in volume from 1 to 13 weeks by 29 %, at which time it had achieved its greatest volume. Thus, the occipital lobe was closer to its maximal volume at birth than any other neocortical region. As indicated above, the occipital lobe then underwent a 16 % decrease in volume through 5 years of age. For cortical areas, the amount of postnatal gain in volume was related to the duration of positive growth. Thus, the frontal and temporal lobes, which had the largest increases in volume, had positive volume changes through 3 years of age. Whereas, the parietal lobe, which had a lower postnatal volume gain, did not show positive gains beyond 6 months. Interestingly, the corpus callosum tripled in size between 1 week and 5 years.

For the profile that we have labeled subcortical structures that contains among other regions the striatum, diencephalon, amygdala and hippocampus, the volume increased by 75 %. The brain stem, which includes the pons and medulla, increased in volume nearly threefold. Finally, the cerebellum increased in volume by 112 % between 1 week and 5 years.

### Hemispheric differences

**Fig. 6:**
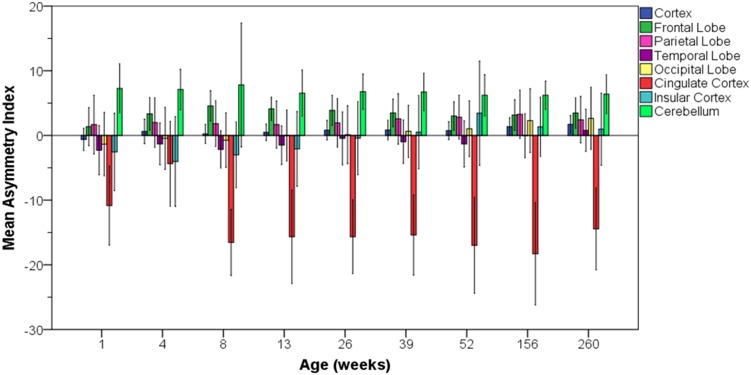
Asymmetry index (±2 standard deviations) for regional brain volumes. Positive values indicate left greater than right hemispheric volume. Regions include cerebral cortex (*dark blue*), frontal lobe (*dark green*), parietal lobe (*magenta*), temporal lobe (*violet*), occipital lobe (*yellow*), cingulate cortex (*red*), insular cortex (*light blue*), cerebellum (*light green*)

To evaluate whether there was a consistent laterality to the volume of brain regions during development, we computed an asymmetry index for each brain region (left volume + right volume/average of left and right volume), with the caveat that the segmentation atlas was asymmetric and not hemispherically mirrored. Hemispheric differences were computed for all bilateral structures at each time point (Fig. 6). For the cerebrum as a whole, a left volume greater than right volume asymmetry was detectable from 4 weeks of age (*t* = 3.151, *p* = 0.004). The asymmetry index was 0.61 % (±0.95 %) at 4 weeks of age and increased to 1.72 % (±0.69 %) by 5 years. By 5 years of age, all cortical regions were larger in the left hemisphere than in the right hemisphere except for the cingulate cortex. The cingulate cortex was 14.46 % (±3.16 %) larger on the right side at 5 years of age and, in fact, was heavily right biased from 1 week of age. The frontal and parietal lobes consistently displayed a leftward asymmetry from 1 week onward. The temporal, insular and occipital regions initially demonstrated slightly larger volumes in the right hemisphere but by 3 years of age all had shifted to larger volumes in the left hemisphere.

Similar to the cerebral cortex, the cerebellum showed a clear leftward volume asymmetry from the earliest time point (*t* = 18.718, *p* < 0.001) which was stable through 5 years of age. The cerebellum was 6.78 % (±2.19 %) larger on the left at 5 years of age—substantially greater laterality than for the cerebrum as a whole.

### Somatic growth

**Fig. 7:**
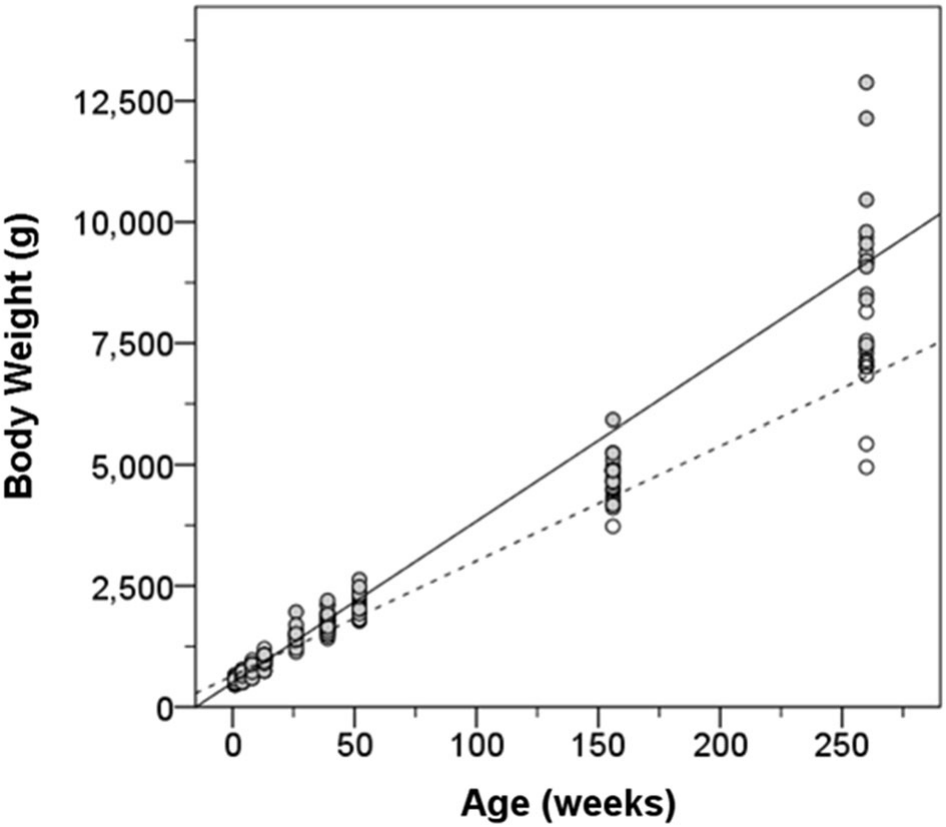
The trajectory of body weight growth for male (*solid*) and female (*dashed*) subjects from 1 to 260 weeks of age. Each *circle* represents one animal subject. Male body weight increases faster than female weight and both begin to slow near 3 years of age

Somatic growth was characterized by weight measurements collected through 5 years of age, as well as measures of various body lengths and circumferences through 3 years of age (Table 9). Partially due to the animal selection criterion for birth weight greater than 450 g, body weight ranged from 460 to 660 g at 1 week of age (Fig. 7). Body weight did not significantly differ by sex until 39 weeks of age, at which time male body weight exceeded female body weight on average (repeated measures ANOVA: 39 weeks, *F* = 8.571, *p* = 0.008; 52 weeks, *F* = 6.438, *p* = 0.019; 156 weeks, *F* = 12.186, *p* = 0.002; 256 weeks, *F* = 29.361, *p* < 0.001). The increasingly divergent trajectories in body weight were demonstrated by a significant age by sex interaction (*F* = 134.5, *p* < 0.001). Therefore, separate linear growth trajectories were estimated for males (33.3 g/week, SE = 0.62) and females (23.7 g/week, SE = 0.48).

**Table 9:** Somatic growth

	Age (weeks)							
	1	4	8	13	26	39	52	156
Female								
Body weight	540 (64)	639 (79)	788 (93)	968 (121)	1423 (120)	1651 (135)	2112 (172)	4411 (341)
Crown-rump length	214 (8)	223 (14)	233 (16)	257 (13)	302 (13)	315 (12)	329 (14)	477 (32)
Head circumference	210 (5)	216 (3)	222 (6)	231 (6)	240 (6)	245 (5)	254 (6)	277 (10)
Bi-parietal diameter	53 (2)	55 (2)	58 (2)	60 (2)	63 (3)	65 (3)	65 (2)	72 (2)
Occipito-frontal diameter	69 (1)	72 (1)	75 (1)	77 (1)	81 (7)	80 (3)	81 (3)	86 (4)
Chest circumference	168 (9)	173.1 (9)	186.4 (13)	201.8 (15)	229.1 (14)	246.0 (13)	263.8 (15)	324.4 (10)
Femur length	65 (2)	70 (3)	75 (3)	81 (3)	97 (4)	102 (3)	112 (4)	170 (12)
Foot length	93 (4)	95 (5)	100 (10)	110 (5)	119 (6)	121 (5)	125 (6)	149 (12)
Hand length	68 (5)	69 (3)	71 (9)	79 (4)	81 (5)	84 (4)	86 (4)	96 (31)
Male								
Body weight	566 (58)	662 (63)	782 (84)	954 (112)	1489 (187)	1872 (214)	2297 (183)	4950 (404)
Crown-rump length	215 (9)	227 (14)	234 (8)	257 (14)	296 (15)	319 (17)	340 (11)	480 (22)
Head circumference	210 (7)	222 (8)	227 (6)	235 (7)	245 (8)	254 (6)	259 (9)	287 (11)
Bi-parietal diameter	54 (2)	57 (2)	59 (2)	60 (2)	65 (2)	66 (2)	66 (3)	74 (3)
Occipito-frontal diameter	71 (2)	73 (2)	75 (2)	77 (3)	80 (3)	82 (3)	83 (3)	90 (4)
Chest circumference	165 (7)	178 (8)	188 (11)	203 (12)	232 (13)	258 (12)	271 (10)	338 (14)
Femur length	67 (7)	71 (2)	76 (4)	81 (7)	96 (3)	106 (4)	114 (4)	182 (7)
Foot length	90 (6)	98 (7)	102 (7)	107 (7)	119 (6)	124 (7)	128 (6)	152 (11)
Hand length	68 (5)	70 (7	74 (5)	76 (5)	84 (6)	89 (5)	89 (4)	106 (10)

Mean (standard deviation) in mm for all measures except body weight in grams

**Fig. 8:**
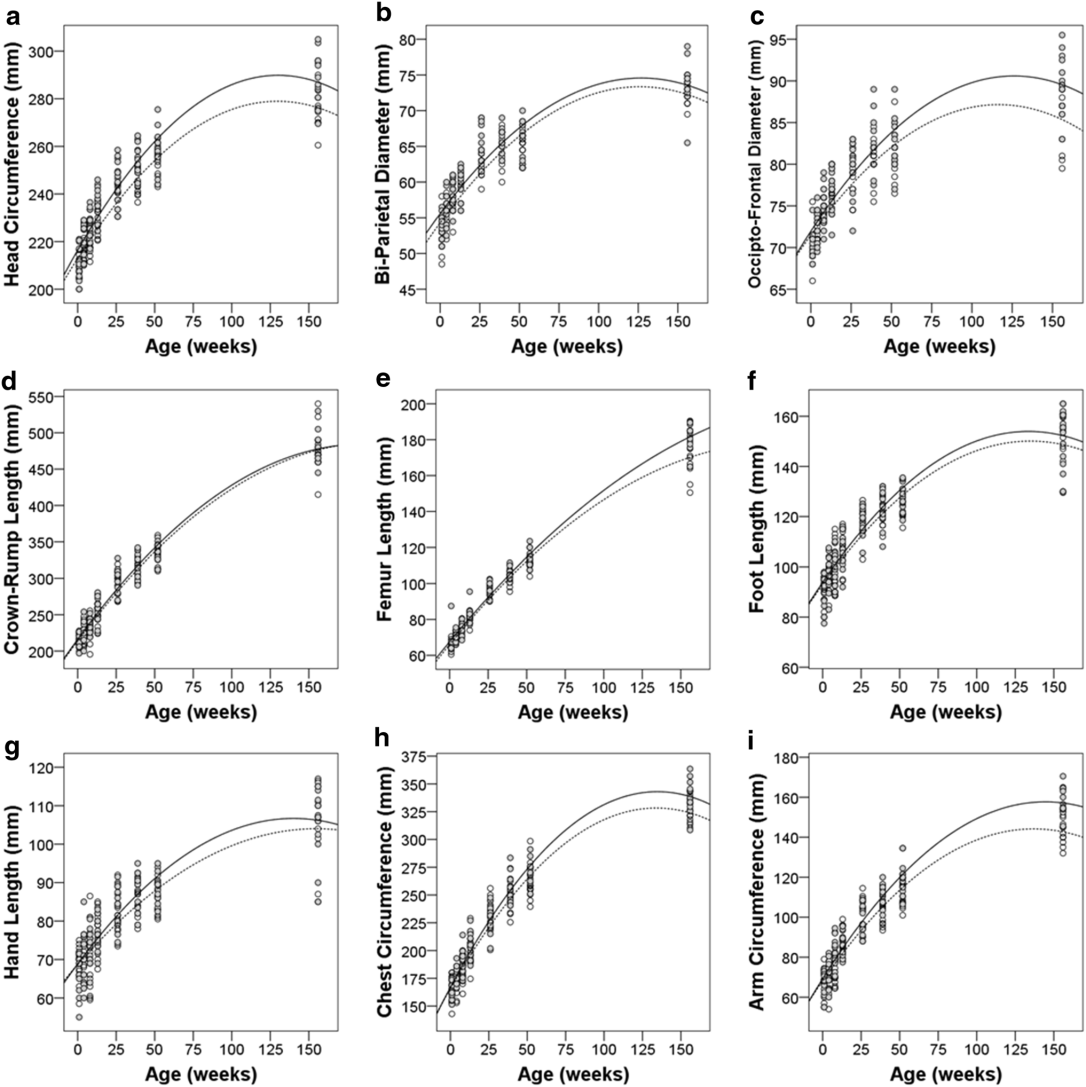
Physical growth parameters for male (*solid*) and female (*dashed*) subjects from 1 to 156 weeks of age. The corresponding quadratic curves are sex-specific estimated growth trajectories. **a** Head circumference, **b** bi-parietal diameter, **c** occipito-frontal diameter, **d** crown-rump length, **e** femur length, **f** foot length, **g** hand length, **h** chest circumference, and **i** arm circumference

Head circumference (HC), as well as bi-parietal (BP) and occipito-frontal (OF) diameters, was used as measures of head size (Fig. 8a–c). Head circumference increased approximately 22 % over the first year of life and increased an additional 8 % from 1 to 3 years. On average, male head circumference was larger than female head circumference (*F* = 4.58, *p* = 0.035). While the temporalis muscles are substantially larger in adult male rhesus monkeys compared to females, this difference was not as pronounced at 3 years of age. Thus, the head circumference difference is due, at least in large part, to a larger skull in the males. Like head circumference, bi-parietal and occipito-frontal diameters showed a rapid increase from 1 to 52 weeks, followed by slower positive change up to 3 years (Fig. 8b, c). Bi-parietal and occipito-frontal diameters tended to be greater in males than females (BP: *F* = 3.4, *p* = 0.073; OF: *F* = 4.1, *p* = 0.049).

Crown-rump length was used as another estimate of body growth (Fig. 8d) that would be similar to height in humans. Crown-rump length increased linearly at a rate of 1.52 mm/week (SE = 0.033) over the first year of life. Male and female infants did not differ in average body length (*F* = 0.319, *p* = 0.576) or in the rate of elongation (*F* = 0.236, *p* = 0.628). Therefore, they did not differ in length (height) at 3 years of age, in contrast to body weight and head circumference.

Femur, hand and foot lengths were used as measures of limb growth (Fig. 8e–g). The limb growth was best characterized with quadratic curves (femur, quadratic term *F* = 81.949, *p* < 0.001; hand, quadratic term *F* = 19.369, *p* < 0.001; foot, quadratic term *F* = 88.675, *p* < 0.001). Femur length was significantly longer in males compared to females (*F* = 7.237, *p* = 0.011). In males, femur length increased at a rate of 0.91 mm/week (SE = 0.032) over the first year, then 0.66 mm/week (SE = 0.018) through 3 years of age. Rate of female femur length growth in the first year of life was 0.89 mm/week (SE = 0.029) and then declined to 0.56 mm/week (SE = 0.035) from 1 to 3 years. Hand length increased by approximately 0.49 mm/week (SE = 0.035) from birth until 39 weeks. The estimate for hand growth rate from 39 to 156 weeks is not presented since this is complicated by medically necessitated digit amputations which occurred in animals after weaning due to conspecific aggression. The rate of foot length growth decreased with age, but was positive between all intervals (1–26 weeks, 1.34 mm/week (SE = 0.14); 26–52 weeks, 1.038 (SE = 0.066); 52–156 weeks 0.24 mm/week (SE = 0.018); *p* < 0.001 for all models). There was no significant effect of sex on hand or foot length.

Chest circumference served as a measure of girth and arm circumference as a measure of muscularity (Fig. 8h, i). Chest circumference fits a linear growth curve with a rate of increase of 0.77 mm/week (SE = 0.029). Likewise, arm circumference increased linearly. The gain in arm circumference was estimated to be 0.43 mm/week (SE = 0.016). Sex differences (male greater than female) in arm and chest circumferences were not significant until 39 weeks of age (chest, *F* = 5.143, *p* < 0.034; arm, *F* = 15.712, *p* = 0.001) and were maintained through 3 years of age.

### Brain growth and body growth

We collected the parameters of physical growth described in the previous section to relate them to brain growth over the first year of life. We have established that body weight gain is predictive of how fast the brain grows over the first year of life (*F* = 12.960, *p* < 0.001). Between-subject differences in absolute brain volume were examined by models that included weight, head circumference, and crown-rump length along with log transformed age and sex. Independently, all three body metrics covaried with brain volume (*p* < 0.01). However, when included within a single model, only head circumference (*F* = 13.730, *p* < 0.001) and body weight (*F* = 15.248, *p* < 0.001) were significant predictors of brain volume up to 1 year of age.

We were interested in determining whether the plateau in cerebral growth that we observed for the 26 to 39 week period was associated with any alterations in somatic growth during this period. We found that body weight gain demonstrated a decrease in weekly rate of growth from 26 to 39 weeks compared to 13 to 26 weeks in both males (13–26: 4.1 ± 1.0 %/week; 26–39: 2.3 ± 1.4 %/week) and females (13–26: 3.6 ± 0.7 %/week; 26–39: 1.7 ± 0.7 %/week). Greater rate of body weight gain resumed from 39 to 52 weeks (males 3.3 ± 1.1 %/week, females 3.2 ± 1.4 %/week). The reduction in body weight gain occurring simultaneously with a plateau in brain growth may be one reason why body weight may be a reliable predictor of brain volume.

### Comparison of cohorts A and B

Cohort B animals were imaged on a 1.5T scanner in part to determine whether volume assessments would be similar when susceptibility artifacts were less of a factor. When we observed the hitherto unreported plateau in total brain growth between 26 and 39 weeks, it became important to attempt an independent replication of this novel finding. Since data were only collected for the time points in the first year of life for cohort B, comparison between the cohorts was restricted to this age range. For the sake of brevity, we have provided complete documentation of brain volumes in the Supplemental Data. Here, we highlight similarities and differences between the cohorts.

Mean birth weight of cohort B (male 528 ± 42 g, female 516 ± 48 g) did not differ from cohort A (male *F* = 3.296, *p* = 0.082; female *F* = 0.596, *p* = 0.448). Body weight only significantly differed between the cohorts in males at 39 (*F* = 6.758, *p* = 0.17) and 52 weeks (*F* = 9.283, *p* = 0.006) at which time cohort A males were heavier.

Despite the different imaging strategies, we did not observe differences in TBV in males. TBV at 1 week was 62,256 mm^3^ (±3879 mm^3^) in cohort A and 59,511 mm^3^ (±4879 mm^3^) in cohort B. At 1 year, TBV had increased to 91,739 mm^3^ (±6114 mm^3^) in cohort A males and to 88,474 mm^3^ (±7269 mm^3^) in cohort B males. TBV in females, however, was significantly lower at 26 (*F* = 9.047, *p* = 0.008), 39 (*F* = 5.940, *p* = 0.019) and 52 weeks of age (*F* = 7.929, *p* = 0.011) in cohort B compared to A.

**Fig. 9:**
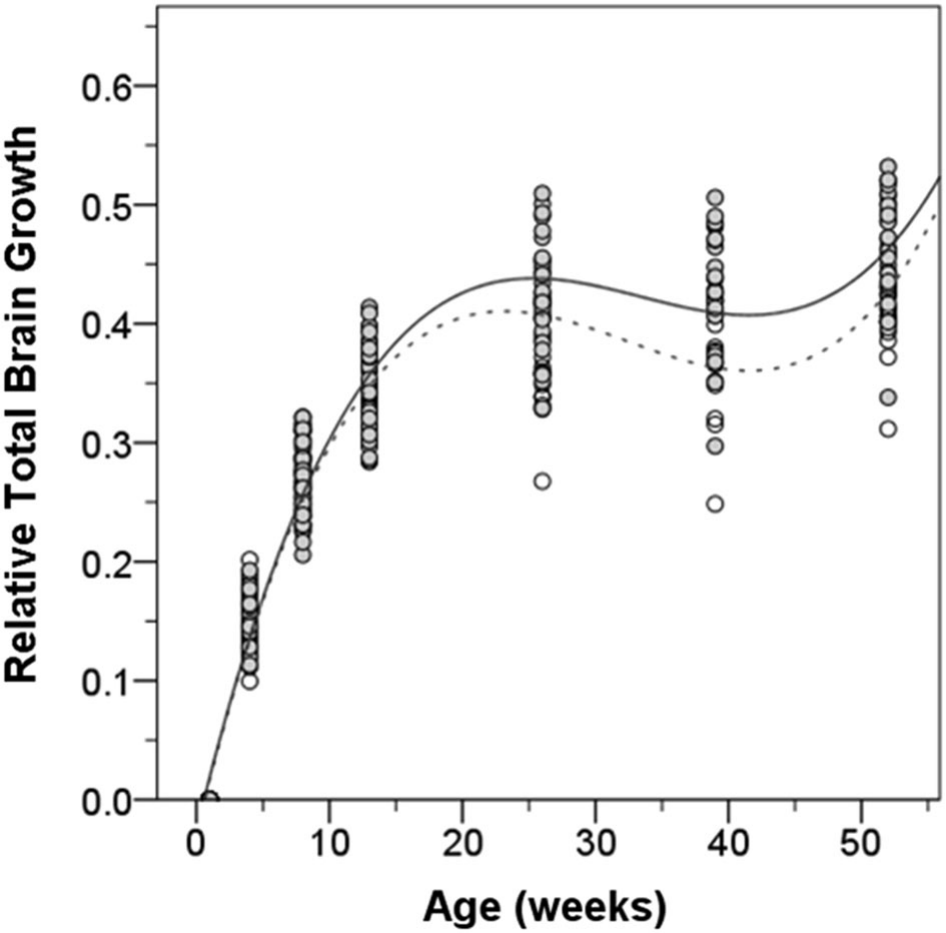
Mean relative brain growth in cohort A (*solid*) and cohort B (*dashed*). Best fit curve and 95 % confidence intervals. The shape of the curve is similar, but cohort B shows less relative gain in brain volume by 1 year of age

When we modeled brain growth and included cohort as a factor, we found that growth rate in cohort B was slower than that of cohort A (*F* = 5.166, *p* = 0.028) (Fig. 9). Over the first year, cohort B showed a 42 % (±5 %) gain in brain volume compared to the 46 % (±5 %) in cohort A. As expected, body weight covaried with brain growth across both cohorts (*F* = 4.586, *p* = 0.033), as well as sex (*F* = 12.950, *p* = 0.001). We then tested whether cohort had an effect in separate models for males and females. Cohort had a significant effect in females (*F* = 7.944, *p* = 0.010), but not males (*F* = 1.027, *p* = 0.326) as males did not differ in TBV. When weight was included in the models along with cohort, weight covaried with brain growth (*F* = 10.016, *p* = 0.002) and the effect of cohort was no longer significant for females (*F* = 0.533, *p* = 0.469) indicating body weight explained the variance in TBV between cohorts. Therefore, the observed cohort difference in TBV growth rate in all subjects was most likely due to weight gain differences in the females.

We were interested in determining whether the plateau in brain growth observed between 26 and 39 months was confirmed in cohort B. We were able to confirm that cohort B showed no significant brain growth between 26 and 39 weeks (*t* = 0.506, *p* = 0.619). On average, and as in cohort A, female brain volume decreased during this interval (−43 ± 250 mm^3^/week) while male volume modestly increased (89 ± 199 mm^3^/week). Beyond TBV, the decrease in volume was also observed in the cerebrum. Different from cohort A, we observed a plateau in cerebellar growth in cohort B during this age range.

In cohort B, male brains were larger than female brains at birth (*F* = 4.369, *p* = 0.05) and grew at a faster rate (*F* = 11.398, *p* = 0.002) such that the relative difference in volume increased with age from 6.9 to 9.6 % by 52 weeks. The relative difference between males and females was more pronounced in cohort B than in cohort A (7.5 % at 52 weeks).

Growth trajectories and asymmetry patterns of cortical regions were amazingly consistent between cohorts A and B (Supplemental Data). In cohort B, male volumes were greater than female volumes for all regions except the frontal lobe (*F* = 0.983, *p* = 0.337) as observed in cohort A. The time course of regional development also paralleled that of cohort A. Frontal and temporal lobe volumes increased through 52 weeks, whereas parietal lobe volume peaked at 26 weeks. The occipital lobe showed the same unusual growth curve relative to other cortical areas. Growth continued rapidly until 13 weeks and then decreased through the remainder of the first year of life. Corpus callosum volume doubled during the first year as in cohort A.

We were also able to confirm that there is generally an overall leftward hemispheric asymmetry of the cortex which was detectable in cohort B at 4 weeks and later. At 1 week of age, frontal and parietal lobes showed leftward asymmetry. In contrast to cohort A, the occipital lobe in cohort B showed leftward asymmetry from week 1 and onward. Rightward asymmetry gradually decreased with age in the temporal lobe. As in cohort A, a rightward asymmetry of the cingulate cortex was consistent over the first year (−15.3 ± 6.5 %) in cohort B. The insula varied in direction of asymmetry across time points in cohort B. The cerebellar volume was greater in the left hemisphere at all ages in cohort B (7.0 ± 2.2 %). The asymmetry determination in cohort B carries the same limitations as in cohort A since they are generated from the same original segmentation atlas.

## Discussion

The data in this paper provide the most comprehensive magnetic resonance imaging assessment of postnatal brain development of naturalistically reared rhesus monkeys to date. A total of 49 rhesus monkeys (25 male and 24 female) were longitudinally evaluated with magnetic resonance imaging at seven time points during the first year of life. A subset of the animals received additional MRI scans at 3 years and 5 years of age. These data have established age-specific T1-weighted segmentation templates from the early postnatal through young adult stages of the rhesus macaque lifespan.

### Major findings

We found that TBV at 1 week was approximately 64 % of that of the adult. Brain volume was larger in male rhesus monkeys compared to females. The extent and trajectory of enlargement varied from cortical region to region. The frontal and temporal lobes showed the largest postnatal increase in volume whereas the occipital lobe showed the smallest increase. The occipital lobe demonstrated the most idiosyncratic developmental profile. It reached maximal size by 13 weeks of age and then decreased by 16 % at 5 years of age. Interestingly, (Bourgeois and Rakic 1993) had shown some time ago that the peak cortical thickness in the monkey occipital lobe was also reached around 3 months of age. This was also the time point of peak density of synapses (30 per 100 µm^2^). Thereafter, between 4.5 and 20 years of age, synaptic density decreased to 20 synapses per 100 µm^2^. Thus, our MRI findings are consistent with the time course of synapse elimination demonstrated by electron microscopic methods.

Of the allometric measurements that were taken, body weight appeared to be most predictive of brain volume growth. In general, there was a leftward asymmetry in brain size. Total brain size, cerebellar size and many cortical regions were larger on the left side than on the right. The most striking exception to this was the cingulate cortex which was substantially right biased through all time points.

Perhaps the most novel of this study’s discoveries is a unique period of no net growth between 6 and 9 months of age. For the female subjects, there was actually a decrease in brain size during this period whereas for the males there was a pronounced slowing of positive brain growth. Interestingly, we also noted a slowing of body growth during this period. It is between 4 and 7.5 months of age that the mother typically weans the infant and restricts access to nursing (Vandeleest and Capitanio 2012). The animals are well fed during this period and there is no indication that the weaning leads to malnourishment. It is more likely that the stress of independence and the greater physical activity that occurs during this period may contribute to the decreased body growth. It is possible, therefore, that the slowed brain growth may be a consequence of the overall slowing of body growth. However, analogous patterns are also observed in human brain development in the absence of such environmental stressors affecting body growth. Brain weight and volume studies have reliably demonstrated that the rate of brain growth slows dramatically between 3 and 4 years of age (Dekaban 1978; Pfefferbaum et al. 1994), which is analogous to the period around 9 months in the rhesus monkey. Also at this time in the human, the close correlation between rate of brain and body growth diminishes, as seen here with the rhesus monkey (Dekaban 1978). An alternative explanation is that progressive neurodevelopmental processes such as axonal outgrowth and dendritic maturation are transiently outpaced by regressive processes such as axonal retraction and programmed cell death. Since all these processes are taking place concurrently (LaMantia and Rakic 1990), it is possible that the balance shifts in the negative direction during periods of slow growth. The resolution of this issue will necessitate additional developmental analyses during this critical age range.

A strength of this report is that all findings have been shown in two independent cohorts of rhesus monkeys (cohort A as discovery and cohort B as replication). All the monkeys were born and raised in naturalistic, outdoor enclosures that provide a reasonably normal social environment for rhesus development. This environment provides perhaps the closest situation to animals in the wild that nonetheless allows scientific investigation. If anything, the animals in this environment received better health care and nutrition than they would receive in the wild. The fact that very similar findings were achieved with cohorts born 2 years apart and scanned on two different MRI platforms attests to the robustness of the findings. These data thus provide a solid basis for future studies of abnormal brain development due to fetal or early postnatal manipulations. As indicated previously, these data are freely available to the neuroscience community.

### Comparison with previous studies

The initial longitudinal MRI study of rhesus macaque brain growth was carried out by Malkova et al. (2006). Despite differences in sample size and rearing conditions, many of the observations are quite similar across studies. For example, we observed an increase in TBV from 1 week to 3 years of about 56 % and they observed an increase of 56 % from 1 week to 4 years, with little change between 3 and 4 years. Interestingly, Malkova and colleagues reported reductions in brain size from 5 to 8 months of age, followed by a rebound at 12 months. This period overlaps with the 6–9 month plateau in brain growth that we observed. In the recent study by Liu et al. (2015), 14 male rhesus monkeys underwent longitudinal MRI scanning from 6 to 16 months. However, 10 of the animals were treated with PCP and thus the results are not comparable to those from the current two cohorts of untreated animals.

In addition to TBV, we evaluated the trajectories of development for several cortical regions of interest. We found that these varied in the age at which peak volume was reached as well as the total percent volume gained. These ROI-based data suggest a spatially varying developmental course for the cerebral cortex, which has been observed previously in rhesus monkey and human imaging studies (Gogtay et al. 2004; Knickmeyer et al. 2010). In comparable cortical regions, Knickmeyer et al. (2010) showed that white matter increases during the juvenile and adolescent periods (1–5 years) in all areas except the occipital lobe. Concurrently, they observed little change in gray matter volume outside of the frontal lobe over the same period. We found that the volume of the occipital and parietal cortices did not significantly increase after 1 year of age, while frontal and temporal cortices did. This is consistent with the notion that cortical regions serving low order functions (e.g., occipital lobe) stabilize earlier than association regions (e.g., frontal lobe) (Gibson 1991).

### Sexual dimorphism in brain size

Even though males and females weighed approximately the same at birth, the female brain was smaller and remained smaller throughout the analysis period. Thus, males had greater magnitude of postnatal growth than females. Sexually dimorphic differences in brain size observed at birth may be due to factors such as prenatal gonadal hormonal milieu (Arnold et al. 2004; Resko and Roselli 1997) or other epigenetic factors such as imprinting (Gregg et al. 2010). Our findings are very similar to those of Joffe et al. (2005) who measured head circumference and femur length in 116 rhesus monkeys from birth to 6 months by the same methods used in this study. Infant male monkeys had larger head circumferences than females and this difference was not accounted for by variation in body length. When we compared brain growth with other allometric features, we found that the rate of brain growth was associated with the rate of increase in body weight, but not with change in body length.

The time course of postnatal brain growth of males and females also differed; it appeared that female brain development was accelerated relative to males. For example, the peak volume of some regions such as the frontal lobe occurred sooner in females than in males. Another example of this is that the female animals showed a net volume decrease from 6 to 9 months, while males only showed evidence of slowed growth. One possibility is that male volume does actually decline but at a time (for example between 10 and 11 months) in between our series of MRI scans. Investigation on a finer time scale would be required to better address time course differences by sex.

### Hemispheric asymmetry

We observed a consistent leftward cortical and cerebellar asymmetry that emerged by 6 months of age. Asymmetry in the rhesus monkey brain is not consistently reported (Iturria-Medina et al. 2011; Short et al. 2014). It is likely that the current larger sample size and the longitudinal design of our study enabled these differences to be more consistently observed. We have shown that structural laterality of some regions (e.g., insula, temporal cortex, and occipital cortex) gradually changes with age, while others (e.g., parietal cortex, frontal cortex, and cerebellum) have established leftward asymmetry by birth.

However, error in assessing asymmetry may be caused by bias in midline boundary assignments, particularly for the cingulate cortex that is relatively small and has a large midline boundary. Further, hemispheric rater bias in the original user-guided segmentation atlas (Knickmeyer et al. 2008) may have also contributed to general leftward asymmetry (Maltbie et al. 2012). If these were the sole reasons for the leftward asymmetry reported here, then left greater than right volumes would be present at all ages, especially for structures with a midline boundary, and this is not the case (e.g., occipital cortex). However, greater accuracy in the absolute differences in hemispheric volumes would be improved by replicating volumetric asymmetry analysis with volumes generated by automated segmentation based on a mirrored atlas (Maltbie et al. 2012). The overall brain findings are, however, consistent with our previous finding of leftward bias for the volume of the hippocampal formation, based on manual tracing with high inter- and intra-rater reliability (Hunsaker et al. 2014). These manual hippocampal tracings were also replicated on mirrored images, to account for user-guided bias, as part of the validation process of a semi-automated hippocampal segmentation method (Hunsaker and Amaral 2014).

In adult humans, the left hemisphere is larger than the right, with some regional exceptions. However, this regional volumetric asymmetry is not apparent in the early postnatal period (Gilmore et al. 2007; Wada et al. 1975). While the occipital lobe shows clear leftward asymmetry, the prefrontal cortex does not (Gilmore et al. 2007). Though Li et al. (2014a, b) have identified persistent structural asymmetries in the perisylvian region, superior temporal sulcus, and supramarginal gyrus in infant brains. Asymmetry gradually increases with age, such that the characteristic leftward asymmetry is found in older children and in right-handed individuals (for review Toga and Thompson 2003).

### Developmental processes underlying volume changes

The time courses of monkey and human neurogenesis, synaptogenesis, and myelination have been well characterized by fundamental works over the past 50 years. In the monkey, cortical neurogenesis is complete near the beginning of the third trimester of gestation and would, therefore, not contribute to postnatal volume growth. However, postnatal synaptic density in the macaque brain undergoes dynamic postnatal changes (Bourgeois et al. 1994; Bourgeois and Rakic 1993; Zecevic et al. 1989), which may contribute to volumetric changes. Rakic and colleagues have presented evidence that synaptogenesis in the rhesus monkey rapidly increases postnatally through 2 months of age across multiple cortical regions. Regions differ, however, in the rate of decline in puberty and early adulthood. Specifically, primary visual cortex shows an earlier and sharper decline in synaptic density after 2 years (Bourgeois and Rakic 1993), while prefrontal cortex does not show net synapse elimination until 3 years (Bourgeois et al. 1994). A similar spatiotemporal pattern of synaptogenesis was reported in the human brain by Huttenlocher and Dabholkar (1997) between primary auditory cortex and prefrontal cortex, in which net elimination of synapses began in early puberty in auditory cortex and later in the prefrontal cortex (Huttenlocher and Dabholkar 1997). Synaptic pruning may be one component of the slight volume declines observed from 3 to 5 years of age.

### Comparison to human MRI studies

Postnatal human brain growth shows similarities and differences with the rhesus monkey. The most distinct difference is that the rhesus monkey brain has relatively less postnatal growth (Holt et al. 1975; Kerr et al. 1969). This difference is underlined by comparison of the volume trajectories from our dataset to a recent human MRI study of brain volume over the first 2 years of life (Knickmeyer et al. 2008). While the rhesus brain increases by 50 % over the first year of life, the human brain increases 100 % in volume over the same period. Differences in regional brain growth, however, are preserved across species. The percent gain in cerebellum volume, for example, was greater than the cerebrum in both the monkey and human. The age range we studied spans the pubertal period and by this stage in the human, regional brain volumes approach their peaks (Lenroot and Giedd 2006); we observed a similar pattern in the rhesus monkey. Either regional volumes were no longer changing (e.g., cingulate cortex) or the rates of change slowed substantially (e.g., temporal lobe). This time course suggests that although the neonatal rhesus monkey brain is more developed than the neonatal human brain, postnatal maturation is protracted in both species through puberty.

### Limitations of the current study

While the current study is the largest, longitudinal, developmental MRI study of the rhesus monkey to date, it included only 45 animals. These animals, though, were maternally reared in a semi-naturalistic setting and thus are likely representative of normal rhesus monkey development. One potential concern is that the removal of the animals from the enclosure and exposure to propofol and ketamine on up to nine occasions in which they were imaged may have introduced an unusual stress to the cohort, which may have altered brain development. We do not believe that this is a serious concern since the animals in the outdoor enclosures are highly adapted to the capture and anesthesia that is part of their routine health evaluations. It would be of interest to determine, however, if other rearing protocols appreciably affect the trajectory of brain development that we have observed. Another issue is that due to the difficulty in reliably segmenting gray and white matter at very early ages, we have not studied the development of these two compartments independently.

### Future directions

The present report is a starting point for more extensive analyses into the relationship between structural development of the brain and behavioral development of the rhesus monkey. The image database will be made available to the scientific community for the exploration of other questions. The social and psychomotor development of these cohorts has also been carefully documented, which can be used to test correlations between brain and behavior. We intend to document correlations between the emergence of species-typical behaviors and brain development in future publications.

## Electronic supplementary material

Below is the link to the electronic supplementary material.

Supplementary material 1 (DOCX 47 kb)
