# Supplementary material for: Longitudinal analysis of the developing rhesus monkey brain using magnetic resonance imaging: birth to adulthood
Source: Brain Struct Funct. 2015 Jul 10;221:2847–71. doi: 10.1007/s00429-015-1076-x (PMC4884209; doi:10.1007/s00429-015-1076-x)
Supplement: Supplementary file 1 — Supplementary material 1 (DOCX 47 kb) [file 429_2015_1076_MOESM1_ESM.docx]

Electronic Supplemental Material

Title: Longitudinal analysis of the developing rhesus monkey brain using magnetic resonance imaging: birth to adulthood

Authors: Julia A. Scott^1,2^, David Grayson^2^, Evan Fletcher^1^, Aaron Lee^2^, Melissa Bauman^2^, Cynthia Mills Schumann^2^, Michael H. Buonocore^3^, David G. Amaral^2^

^1^Department of Neurology, University of California, Davis

^2^Department of Psychiatry and Behavioral Sciences, the MIND Institute, University of California, Davis

^3^Department of Radiology, University of California, Davis

Corresponding author:

David G. Amaral, Ph.D.

University of California, Davis

The MIND Institute

2825 50th Street

Sacramento, CA 95817

Telephone: (916) 703-0225

Fax: (916) 703-0287

e-mail: dgamaral@ucdavis.edu

Table S1. Cohort B Bilateral Brain Volumes^a^

|  | **Age (weeks)** | | | | | | |
| --- | --- | --- | --- | --- | --- | --- | --- |
|  | **1** | **4** | **8** | **13** | **26** | **39** | **52** |
| **Female** |  |  |  |  |  |  |  |
| **Total Brain** | 57,352 | 66,056 | 72,968 | 77,316 | 78,729 | 78,171 | 80,350 |
|  | (3,772) | (4,329) | (4,247) | (5,280) | (5,481) | (5,190) | (5,378) |
| **Cerebrum** | 52,181 | 59,939 | 65,858 | 69,534 | 70,058 | 69,142 | 70,916 |
|  | (3,292) | (3,766) | (3,646) | (4,590) | (4,665) | (4,477) | (4,590) |
| **Subcortical Structures** | 7,042 | 7,753 | 8,450 | 9,028 | 9,614 | 10,017 | 10,492 |
|  | (385) | (481) | (507) | (671) | (670) | (691) | (813) |
| **Corpus Callosum** | 314 | 403 | 505 | 592 | 716 | 762 | 766 |
|  | (40) | (44) | (48) | (62) | (72) | (82) | 979) |
| **Brain Stem** | 915 | 1,024 | 1,148 | 1,266 | 1,457 | 1,630 | 1,740 |
|  | (100) | (101) | (122) | (148) | (182) | (179) | (194) |
| **Male** |  |  |  |  |  |  |  |
| **Total Brain** | 61,475 | 69,862 | 77,866 | 82,184 | 84,907 | 86,045 | 88,474 |
|  | (5,090) | (5,454) | (5,982) | (6,422) | (5,652) | (6,590) | (7,269) |
| **Cerebrum** | 55,986 | 63,379 | 70,266 | 73,838 | 75,542 | 76,235 | 78,143 |
|  | (4,643) | (4,884) | (5,349) | (5,716) | (5,042) | (5,925) | (6,489) |
| **Subcortical Structures** | 7,725 | 8,395 | 9,280 | 9,929 | 10,652 | 11,209 | 11,775 |
|  | (646) | (691) | (803) | (848) | (850) | (905) | (1,036) |
| **Corpus Callosum** | 335 | 454 | 595 | 679 | 835 | 905 | 908 |
|  | (32) | (54) | (73) | (83) | (92) | (107) | (106) |
| **Brain Stem** | 989 | 1,097 | 1,252 | 1,393 | 1,611 | 1,791 | 1,938 |
|  | (92) | (98) | (112) | (136) | (125) | (147) | (165) |

**^a^** Mean (standard deviation) in mm^3^

Table S2. Cohort B Regional Brain Volumes, Right Hemisphere^a^

|  | **Age (weeks)** | | | | | | |
| --- | --- | --- | --- | --- | --- | --- | --- |
|  | **1** | **4** | **8** | **13** | **26** | **39** | **52** |
| **Female** |  |  |  |  |  |  |  |
| **Frontal Lobe** | 6,176 | 7,111 | 7,896 | 8,468 | 8,776 | 8,769 | 9,093 |
|  | (453) | (521) | (499) | (678) | (741) | (597) | (654) |
| **Parietal Lobe** | 4,442 | 5,138 | 5,698 | 5,942 | 5,963 | 5,737 | 5,738 |
|  | (292) | (292) | (333) | (364) | (411) | (423) | (330) |
| **Temporal Lobe** | 5,474 | 6,308 | 7,023 | 7,615 | 7,883 | 7,812 | 7,983 |
|  | (376) | (453) | (455) | (568) | (598) | (583) | (579) |
| **Occipital Lobe** | 5,440 | 6,462 | 7,026 | 7,140 | 6,547 | 6,190 | 6,129 |
|  | (501) | (589) | (610) | (653) | (528) | (605) | (585) |
| **Cingular Cortex** | 740 | 868 | 866 | 902 | 892 | 886 | 932 |
|  | (62) | (77) | (64) | (85) | (94) | (68) | (87) |
| **Insular Cortex** | 314 | 352 | 358 | 374 | 372 | 369 | 373 |
|  | (29) | (32) | (29) | (33) | (23) | (22) | (28) |
| **Cerebellum** | 2,057 | 2,426 | 2,807 | 3,054 | 3,354 | 3,420 | 3,611 |
|  | (252) | (286) | (314) | (343) | (369) | (350) | (378) |
| **Male** |  |  |  |  |  |  |  |
| **Frontal Lobe** | 6,524 | 7,463 | 8,324 | 8,854 | 9,301 | 9,591 | 10,008 |
|  | (532) | (574) | (594) | (655) | (519) | (660) | (760) |
| **Parietal Lobe** | 4,750 | 5,409 | 6,022 | 6,340 | 6,461 | 6,387 | 6,443 |
|  | (425) | (484) | (506) | (536) | (496) | (553) | (598) |
| **Temporal Lobe** | 5,852 | 6,668 | 7,503 | 8,022 | 8,505 | 8,582 | 8,768 |
|  | (490) | (510) | (541) | (597) | (591) | (755) | (835) |
| **Occipital Lobe** | 5,831 | 6,736 | 7,401 | 7,460 | 7,022 | 6,740 | 6,657 |
|  | (576) | (595) | (686) | (686) | (558) | (578) | (579) |
| **Cingular Cortex** | 818 | 932 | 940 | 971 | 971 | 981 | 1,025 |
|  | (73) | (89) | (92) | (93) | (95) | (93) | (115) |
| **Insular Cortex** | 340 | 372 | 383 | 404 | 394 | 405 | 400 |
|  | (37) | (40) | (47) | (52) | (51) | (47) | (42) |
| **Cerebellum** | 2,174 | 2,558 | 2,956 | 3,243 | 3,562 | 3,650 | 3,898 |
|  | (229) | (262) | (286) | (324) | (287) | (333) | (407) |

**^a^** Mean (standard deviation) in mm^3^

Table S3. Cohort B Regional Brain Volumes, Left Hemisphere^a^

|  | **Age (weeks)** | | | | | | |
| --- | --- | --- | --- | --- | --- | --- | --- |
|  | **1** | **4** | **8** | **13** | **26** | **39** | **52** |
| **Female** |  |  |  |  |  |  |  |
| **Frontal Lobe** | 6,067 | 6,855 | 7,526 | 8,139 | 8,479 | 8,432 | 8,866 |
|  | (470) | (530) | (494) | (653) | (732) | (611) | (594) |
| **Parietal Lobe** | 4,298 | 4,967 | 5,470 | 5,740 | 5,757 | 5,514 | 5,659 |
|  | (270) | (300) | (336) | (394) | (377) | (385) | (316) |
| **Temporal Lobe** | 5,647 | 6,432 | 7,167 | 7,640 | 7,908 | 7,863 | 8,073 |
|  | (389) | (462) | (489) | (558) | (579) | (593) | (551) |
| **Occipital Lobe** | 5,376 | 6,416 | 6,957 | 7,052 | 6,415 | 6,114 | 6,091 |
|  | (460) | (588) | (569) | (643) | (503) | (568) | (631) |
| **Cingular Cortex** | 848 | 913 | 1,049 | 1,115 | 1,095 | 1,079 | 1,093 |
|  | (72) | (61) | (67) | (97) | (97) | (85) | (105) |
| **Insular Cortex** | 318 | 363 | 372 | 380 | 358 | 360 | 394 |
|  | (24) | (29) | (18) | (26) | (20) | (31) | (29) |
| **Cerebellum** | 1,884 | 2,263 | 2,650 | 2,869 | 3,144 | 3,218 | 3,316 |
|  | (240) | (283) | (297) | (338) | (379) | (337) | (308) |
| **Male** |  |  |  |  |  |  |  |
| **Frontal Lobe** | 6,479 | 7,250 | 8,027 | 8,637 | 9,055 | 9,316 | 9,660 |
|  | (511) | (518) | (522) | (598) | (508) | (676) | (710) |
| **Parietal Lobe** | 4,580 | 5,278 | 5,829 | 6,131 | 6,246 | 6,198 | 6,319 |
|  | (439) | (447) | (464) | (515) | (508) | (591) | (607) |
| **Temporal Lobe** | 6,023 | 6,805 | 7,650 | 8,099 | 8,466 | 8,645 | 8,790 |
|  | (565) | (565) | (620) | (691) | (651) | (788) | (756) |
| **Occipital Lobe** | 5,794 | 6,733 | 7,378 | 7,410 | 6,933 | 6,609 | 6,677 |
|  | (483) | (556) | (623) | (642) | (485) | (471) | (632) |
| **Cingular Cortex** | 925 | 953 | 1,122 | 1,174 | 1,156 | 1,175 | 1,195 |
|  | (110) | (100) | (125) | (129) | (99) | (115) | (113) |
| **Insular Cortex** | 345 | 385 | 407 | 407 | 381 | 396 | 426 |
|  | (29) | (38) | (51) | (41) | (48) | (51) | (48) |
| **Cerebellum** | 1,991 | 2,376 | 2,797 | 3,030 | 3,357 | 3,464 | 3,587 |
|  | (204) | (270) | (306) | (322) | (312) | (339) | (357) |

**^a^** Mean (standard deviation) in mm^3^

Table S4. Cohort B Hemispheric Asymmetry Index^a^

|  | **Age (weeks)** | | | | | | |
| --- | --- | --- | --- | --- | --- | --- | --- |
|  | **1** | **4** | **8** | **13** | **26** | **39** | **52** |
| **Cortex** | 0.0% | 0.9% | 0.8% | 0.9% | 1.3% | 1.2% | 0.5% |
|  | (0.8%) | (0.7%) | (0.7%) | (0.8%) | (0.7%) | (0.7%) | (0.8%) |
| **Frontal Lobe** | 1.2% | 3.3% | 4.2% | 3.1% | 3.0% | 3.4% | 3.0% |
|  | (1.3%) | (1.1%) | (1.0%) | (1.3%) | (1.0%) | (1.3%) | (1.1%) |
| **Parietal Lobe** | 3.5% | 2.9% | 3.6% | 3.4% | 3.5% | 3.5% | 1.7% |
|  | (2.5%) | (2.0%) | (2.0%) | (2.0%) | (2.2%) | (2.3%) | (2.0%) |
| **Temporal Lobe** | -3.0% | -2.0% | -1.9% | -0.6% | 0.1% | -0.7% | -0.7% |
|  | (1.7%) | (1.4%) | (1.2%) | (1.6%) | (2.1%) | (1.5%) | (1.9%) |
| **Occipital Lobe** | 0.8% | 0.3% | 0.6% | 0.9% | 1.6% | 1.5% | 0.2% |
|  | (2.6%) | (1.9%) | (1.7%) | (2.0%) | (2.2%) | (3.3%) | (2.6%) |
| **Cingular Cortex** | -12.7% | -3.6% | -18.3% | -19.9% | -18.9% | -18.8% | -15.7% |
|  | (3.8%) | (3.7%) | (3.2%) | (3.7%) | (4.2%) | (3.6%) | (3.5%) |
| **Insular Cortex** | -1.5% | -3.3% | -5.1% | -1.4% | 3.6% | 2.4% | -5.9% |
|  | (4.3%) | (3.5%) | (5.4%) | (5.9%) | (5.0%) | (5.5%) | (4.1%) |
| **Cerebellum** | 8.8% | 7.3% | 5.7% | 6.6% | 6.3% | 5.7% | 8.3% |
|  | (1.6%) | (1.8%) | (2.2%) | (1.8%) | (1.9%) | (1.7%) | (2.3%) |

^a^Positive values left volume greater than right volume. Mean (standard deviation).

Table S5. Cohort B Somatic Growth^a^

|  | **Age (weeks)** | | | | | | |
| --- | --- | --- | --- | --- | --- | --- | --- |
|  | **1** | **4** | **8** | **13** | **26** | **39** | **52** |
| **Female** |  |  |  |  |  |  |  |
| **Body Weight** | 515 | 583 | 709 | 845 | 1,261 | 1,548 | 1,926 |
|  | (48) | (60) | (84) | (91) | (253) | (214) | (218) |
| **Head Circumference** | 207 | 213 | 222 | 227 | 232 | 244 | 249 |
|  | (6) | (8) | (4) | (6) | (9) | (6) | (6) |
| **Crown-Rump Length** | 204 | 215 | 224 | 245 | 281 | 309 | 328 |
|  | (14) | (14) | (10) | (13) | (22) | (23) | (18) |
| **Bi-parietal Diameter** | 52 | 54 | 57 | 59 | 61 | 63 | 64 |
|  | (2) | (2) | (2) | (2) | (2) | (2) | (1) |
| **Orbito-frontal Diameter** | 68 | 71 | 73 | 76 | 79 | 79 | 81 |
|  | (2) | (1) | (2) | (2) | (3) | (3) | (2) |
| **Femur Length** | 64 | 68 | 73 | 79 | 93 | 101 | 113 |
|  | (3) | (4) | (4) | (4) | (7) | (6) | (5) |
| **Male** |  |  |  |  |  |  |  |
| **Body Weight** | 535 | 628 | 765 | 935 | 1,385 | 1,629 | 2,089 |
|  | (43) | (66) | (96) | (120) | (138) | (140) | (142) |
| **Head Circumference** | 214 | 220 | 231 | 234 | 246 | 250 | 254 |
|  | (5) | (7) | (6) | (6) | (5) | (4) | (6) |
| **Crown-Rump Length** | 209 | 209 | 224 | 243 | 287 | 316 | 337 |
|  | (12) | (7) | (11) | (13) | (10) | (17) | (17) |
| **Bi-parietal Diameter** | 52 | 55 | 58 | 60 | 62 | 64 | 66 |
|  | (1) | (2) | (1) | (2) | (1) | (2) | (2) |
| **Orbito-frontal Diameter** | 70 | 73 | 75 | 77 | 80 | 82 | 83 |
|  | (1) | (1) | (2) | (2) | (2) | (1) | (2) |
| **Femur Length** | 66 | 69 | 74 | 80 | 94 | 105 | 116 |
|  | (4) | (3) | (3) | (3) | (4) | (4) | (5) |

**^a^** Mean (standard deviation) in mm for all measures except body weight in grams.
